# From yellow deserts to white mountains: confirmed occurrence and genetic affiliation of *Psammophis schokari* (Forskål, 1775) (Serpentes, Psammophiidae) in Lebanon

**DOI:** 10.3897/zookeys.1268.177920

**Published:** 2026-02-02

**Authors:** Daniel Jablonski, Rami Khashab, Riyad A. Sadek

**Affiliations:** 1 Department of Zoology, Comenius University in Bratislava, Ilkovičova 6, Mlynská dolina 842 15, Bratislava, Slovakia American University of Beirut Beirut Lebanon; 2 Herping Lebanon, Fanar, Metn, Lebanon Comenius University in Bratislava Bratislava Slovakia; 3 School of Arts and Sciences, Lebanese International University, Saloumi Street, Matn, Lebanon Herping Lebanon Fanar Lebanon; 4 Department of Biology, American University of Beirut, Bliss Street, 1107 2020, Beirut, Lebanon Lebanese International University Matn Lebanon

**Keywords:** Colonizations, distribution, historical biogeography, Levant, Middle East, Ophidia

## Abstract

The ecological and biogeographic limits of arid-adapted reptiles in the Eastern Mediterranean remain poorly understood. Here, we document the first confirmed occurrence and genetic affiliation of the desert racer, *Psammophis schokari* (Forskål, 1775), in Lebanon, representing the northern limit of its confirmed distribution in the western Levant, where its presence has long remained uncertain. Seventeen records from 11 localities (17–1,148 m a.s.l.) reveal that the species occupies a wide ecological gradient encompassing lowland agricultural areas, semi-arid foothills, and even seasonally snow-covered sites. Mitochondrial cytochrome *b* sequences place the Lebanese populations within the widespread “Middle Eastern lineage”, yet their haplotypes show a closer affinity to those from North Africa than to currently sampled populations from the southern Levant. This pattern is consistent with historical Afro–Levantine connectivity and suggests that Lebanon may have been reached during one or more Pleistocene dispersal/colonization episodes from northern Africa. The frequent occurrence of individuals in non-desert habitats and even during winter months demonstrates a high degree of ecological flexibility and tolerance to cooler Mediterranean conditions. Our results thus highlight the ability of *P. schokari* to persist and expand beyond typical desert environments and thereby shedding light on the northern biogeographic limits of arid-adapted snakes in the Middle East. This study fills a significant distributional gap for the genus *Psammophis* in the Levant and underscores the need for broader sampling to clarify the species’ past colonization routes and evolutionary history across the region.

## Introduction

Lebanon is a small country located between the central part of the eastern Mediterranean coast and the Syrian desert, divided from north to south by two biogeographically important and high mountain chains, the Lebanon and Anti-Lebanon. Its position, combined with the highly heterogeneous landscape of these mountain chains, is unique: in the past, this setting facilitated both local speciation and the colonization of various evolutionary lineages of small vertebrates from Asia and Africa that converged in this part of Western Asia (In den Bosch et al. 1998). Nevertheless, substantial knowledge gaps in national biodiversity persist, largely due to limited local expertise, ongoing economic constraints, and recurrent socio-political instability.

The herpetofauna of Lebanon is rich and genetically diverse (Jablonski et al. 2023, 2024), comprising several dozen species representing East Mediterranean, steppe-desert, and high-mountain specialists. However, this diversity has not been comprehensively assessed recently, and an updated checklist has been lacking for over two decades (Hraoui-Bloquet et al. 2002). Despite the above-mentioned challenges, several species have been documented in recent years that are either new to science (Jablonski et al. 2023) or newly recorded for the country’s herpetofauna (Dufresnes et al. 2019; Khashab and Jablonski 2022; Tamar and Moravec 2022; Jablonski et al. 2024). This opens an opportunity for field research focusing on species that have been recorded as part of the Lebanese herpetofauna, as well as those that are expected to occur in the country. In this respect, border regions in the north, Anti-Lebanon Mountains, and the southern Mediterranean parts are the least explored and highly promising.

One such species is *Psammophis schokari* (Forskål, 1775), a member of the family Psammophiidae, which possesses one of the broadest geographic distributions among western Palearctic snakes. Its range spans much of North Africa, the Levant, the Arabian Peninsula, and extends eastward into Central Asia and parts of South Asia (see Bar et al. 2021). In Lebanon, however, the presence of this species remained uncertain for a long time. Only two specimens collected from the coastal cities of Sidon and Beirut are reported in the literature (Hraoui-Bloquet 1981), one mentioned to be stored in the American University of Beirut Natural History Museum, Lebanon (probably AUB AR0126 collected in 1961), and the second in the National Museum of Natural History in Paris, France, with no additional information provided.

The first known literature report of the species, however, came from Jan (1863: 90) with the location provided as “Bairut”, stored in the museum in Frankfurt, Germany. Later, Böttger (1880), probably referring to Jan (1863), mentioned the species under the name *Psammophis moniliger* Duméril, Bibron & Duméril, 1854 from “Beyrut,” noting their similarity to populations from northern Africa and Syria, and comparing them with var. *hierosolymitana*, which was then also known from Haifa, Jaffa, and Jerusalem. Later, Werner (1939) argued that since Beirut lies outside desert regions, and the species (under the name *P. sibilans*) was, in his view, strictly desert-dwelling (though it can occur up to 3500 m in Iran; Kamali 2020), the Beirut record appeared highly doubtful. He therefore concluded that Jan’s (not specified but probably the work from 1863) specimen was most likely purchased in Beirut but originated from the southern Levant or the Syrian Desert (Werner 1939).

This scepticism probably explains why no further attention was given to the possible presence of the genus *Psammophis* Fitzinger, 1826 in Lebanon that could provide further connectivity for populations in Syria or northeastern Iraq (Aidek et al. 2023; Jablonski et al. 2025). Despite various herpetological surveys carried out during the 20^th^ century (Müller and Wettstein 1933; Schmidt 1939; Zinner 1967; Esterbauer 1992; In den Bosch et al. 1998), none reported or further mentioned the species in the country, except for Hraoui-Bloquet (1981). Notably, it was also absent from the most recent comprehensive checklist of Lebanese herpetofauna (Hraoui-Bloquet et al. 2002), casting further doubt on its occurrence. On the other hand, several later sources have mentioned this species in Lebanon (e.g. Disi et al. 2001; Sindaco et al. 2013; Bar et al. 2021), but these appear to be repetitions of earlier, uncertain records.

Here, we revisit this issue and provide strong evidence regarding the presence of *P. schokari* in Lebanon, along with extensive distribution data and genetic affiliation of populations from the southern part of the country.

## Material and methods

Between 2018 and 2025, we conducted more than 60 field surveys (1–4 people) in southern and central Lebanon to document amphibian and reptile diversity. Surveys were based primarily on direct observations, carried out mainly from early spring to autumn, with additional visits in winter (November–February). Surveys lasted 1–3 days (mostly single-day) and included nocturnal searches; we spent ≥ 2 h at each site and typically accumulated ~10 h of active searching per survey. These efforts revealed *P. schokari* at multiple localities, representing the first confirmed records for Lebanon since Hraoui-Bloquet (1981). For each observation we recorded date, abundance, age/sex (when determinable), GPS coordinates, nearest village, elevation, habitat type, and relevant field notes. A small number of resident-provided records were included to our database when supported by photographic evidence.

**Figure 1.**
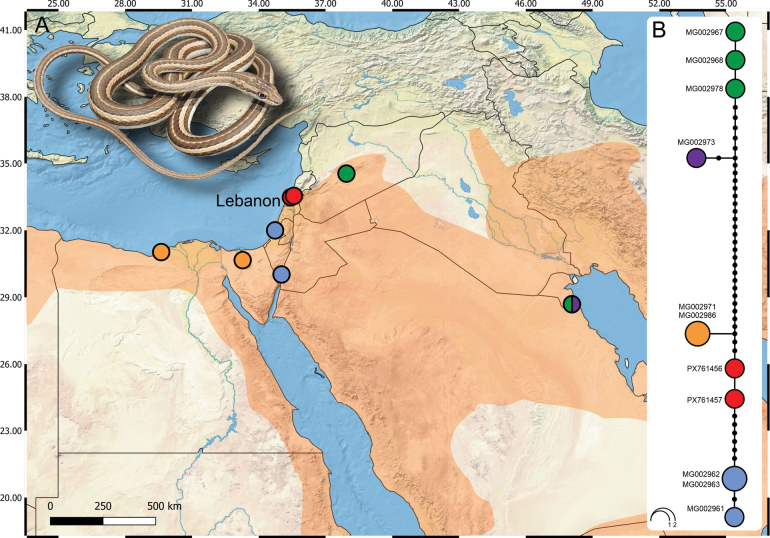
Genetic data of *Psammophis schokari* from Lebanon and the Western Asia. **A**. Geographic distribution of sampled populations with colour-coded localities (Lebanese samples = red; other localities from Gonçalves et al. 2018). Orange shaded area indicates the species’ approximate range in the region; **B**. Statistical parsimony haplotype network of mitochondrial cytochrome *b* showing relationships among haplotypes; circle sizes correspond to sample frequency and colours correspond to sampling localities in panel A. The codes with circles represent GenBank accession numbers. Lebanese haplotypes form a distinct cluster more distant from the southern Levant than from Egypt. Inset photo: *P. schokari* from Kfar Jouz/Kfour, Lebanon (29 March 2022; Photo Rami Khashab).

Together with field data, DNA tissue samples were obtained from two Lebanese populations, particularly from Kfar Jouz/Kfour, Nabatieh Governorate (33.4078°N, 35.4540°E, 352 m a.s.l.) and Ebba, Nabatieh District and Governorate (33.3732°N, 35.4117°E, 260 m a.s.l.) (Table 1; Fig. 1). Based on previous research and the availability of comparative datasets, we generated new mitochondrial sequences of cytochrome *b* marker (cyt *b*) using primers and PCR conditions described by Gonçalves et al. (2018). Total genomic DNA was extracted with the E.Z.N.A.® Tissue DNA Kit, following the manufacturer’s protocol. Newly generated sequences of the species from Lebanon are available in GenBank under the accession numbers PX761456 and PX761457.

**Table 1.** Records of *Psammophis schokari* in Lebanon between 2018 and 2025. Data include map number (corresponding to Fig. 2), field number, date, locality with geographic coordinates, and elevation, age, condition (alive or dead), observed activity, type of evidence, and observer name. DOR = dead on road. For details see Suppl. material 1.

Map number	Field number	Date	Locality	Geographic coordinates (°N, °E)	Elevation (m)	Age	State	Activity	Evidence	Observer
**1**	PSL001	11 September 2018	Qlaileh	33.1944, 35.2461	143	juvenile	Alive	Active, moving	Photo	Mahdi Hardan/Rami Khashab
**2**	PSL002	27 July 2019	Anjar	33.7310, 35.9478	975	adult	Alive	Basking on a rock	Video	Berj Tumberian
**3**	PSL003	29 June 2021	Sohmor	33.5045, 35.6805	868	subadult	Alive	Crossing a road	Photo	Zainab Farhat
**4**	PSL004	29 March 2022	Kfar Jouz/Kfour	33.4075, 35.4547	358	adult	Alive	Active, moving	Photo	Hamza Rida/Rami Khashab
**5**	PSL005	7 June 2022	Kfar Jouz/Kfour	33.4068, 35.4540	347	adult	Dead	DOR	Photo	Hamza Rida
**6**	PSL006	25 May 2023	Kfar Jouz/Kfour	33.4078, 35.4540	352	adult	Alive	Active, moving	Photo	Hamza Rida/Rami Khashab
**7**	PSL007	29 May 2023	Marj El Simah	33.5015, 35.8016	911	adult	Dead	DOR	Video	Tamer Talayeh
**8**	PSL008	2 July 2023	Deir Al Mokhalles	33.5816, 35.4790	429	adult	Dead	Killed by a cat	Photo	Raeda Fares
**9**	PSL009	31 August 2023	Kfar Jouz/Kfour	33.3923, 35.4654	403	subadult	Alive	Under a rock	Photo	Hamza Rida
**10**	PSL010	26 February 2024	Marj El Simah	33.5007, 35.8028	889	adult	Alive	Active, moving (grass habitat)	Photo	Borja de las Heras/Rami Khashab
**11**	PSL011	6 May 2024	Ebba	33.3732, 35.4117	260	adult	Alive	Trapped inside a greenhouse	Photo	Nidaa Hamza/Rami Khashab
**12**	PSL012	15 July 2024	Bedias	33.3249, 35.3051	17	subadult	Dead	Killed by locals	Photo	Rami Khashab
**13**	PSL013	14 September 2024	Kfar Mechki	33.5061, 35.7554	1148	juvenile	Dead	Killed by locals	Photo	Hicham El Zein
**14**	PSL014	15 January 2025	Rihan	33.4469, 35.5635	1050	adult	Alive	Passing a backyard	Photo	Elias Fatouch
**15**	PSL015	25 August 2025	Marj El Simah	33.5055, 35.7950	953	juvenile	Dead	Recorded inside a residential building	Photo	Tamer Talayeh
**16**	PSL016	4 October 2025	Marj El Simah	33.4977, 35.7979	875	adult	Alive	Active in the morning	Photo	Shady Akl/Jad Mershed/Borja de las Heras
**17**	NA	20 November 2022	Sfariyeh	33.5735, 35.4773	165	NA	Alive	Active at the ruin site	NA	Haitham Darazi

For accurate phylogeographic assignment based on mitochondrial sequences, we included material from Gonçalves et al. (2018), particularly for so-called “Middle East lineage”, that were closely related to newly generated Lebanese data. It includes sequences from MG002961–MG002963 (southern Levant), MG002967, MG002968 (Syria), MG002971, MG002986 (Sinai), MG002973, MG002978 (Kuwait). The final sequence alignment (11 sequences, 1098 bp) was analysed with a haplotype-network approach (more effective for presentation of intraspecific evolution in closely related populations than the tree-based visualization) in Hapsolutely (Vences et al. 2024). To visualize the genetic diversity in the geographic context, distribution of the species, and update its known range in Lebanon, we prepared distribution maps using QGIS 3.44 Solothurn (QGIS Development Team 2025; https://qgis.org/).

## Results

The mitochondrial cytochrome *b* sequences group within the widespread “Middle Eastern lineage” of the species, sensu Gonçalves et al. (2018). However, the haplotype network (Fig. 1B) reveals a distinct clustering of Lebanese haplotypes. Surprisingly, the Lebanese samples from two distinct haplotypes show a closer relationship to Egyptian haplotypes (4 mutation steps) than to those from the southern Levant (more than 7), indicating a relatively high degree of differentiation across short geographic distances.

**Figure 2.**
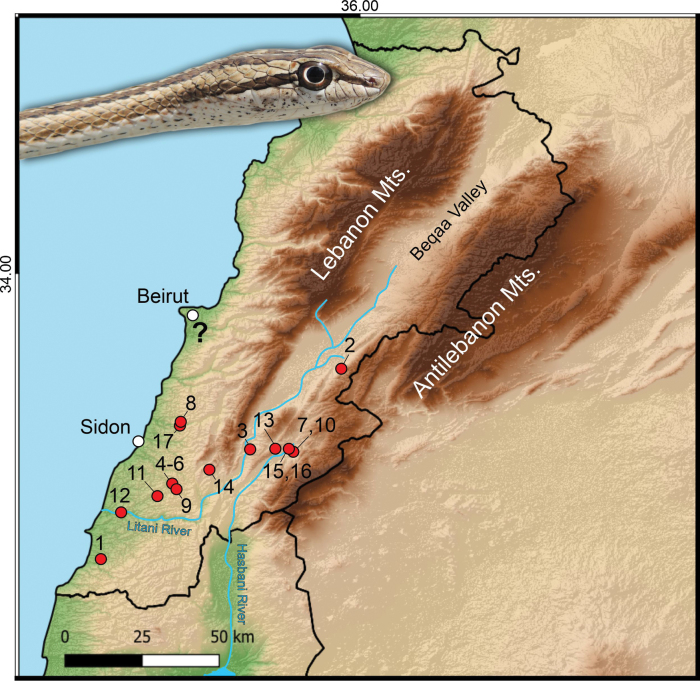
Distribution of *Psammophis schokari* in Lebanon based on records listed in Table 1. Red circles = newly documented localities, white circles = historical data. The question mark indicates historical record from a vicinity of Beirut (Hraoui-Bloquet 1981). Background shows shaded relief with major mountain ranges (Lebanon Mts., Anti-Lebanon Mts.) and the Beqaa Valley. Two rivers, Litani and Hasbani represent possible corridors for the species colonization. Inset photo: live individual of *P. schokari* from Kfar Jouz/Kfour, Lebanon (29 March 2022; Photo Rami Khashab).

We documented 17 records of *P. schokari* in Lebanon between 2018 and 2025, representing 11 localities distributed across the Beqaa Valley and southern Lebanon (Table 1; Figs 1, 2). The records span a broad elevational gradient from 17 m a.s.l. (Bedias, Qasmieh/Litani area, South Governorate) to 1,148 m a.s.l. (Kfar Mechki, Rachaya district, Beqaa Governorate), thus covering both lowland plains and foothill habitats of the Lebanon and Anti-Lebanon ranges. The southernmost and westernmost record originates from Qlaileh, South Governorate, and the northernmost and easternmost confirmed record from Anjar, Beqaa Governorate. Except for the Sfariyeh record, all records were supported by photographs (n = 14) or videos (n = 2). One specimen was deposited in the American University of Beirut Natural History Museum (AUB) under the voucher museum number AUB AR0936. Original photo vouchers of recorded individuals are available in Suppl. material 1.

The first known specimen of this study was a juvenile from Qlaileh (11 September 2018). Because several *P. schokari* appeared simultaneously in a Beirut pet shop, likely imported illegally from Egypt, its native origin was uncertain. The individual died shortly after capture (AUB AR0936). The species’ native presence was confirmed the following year (27 July 2019) by a filmed observation of an adult at Anjar (975 m a.s.l.). A third observation came from Sohmor (29 June 2021), followed by the first well-documented specimen (with obtained DNA sample) from Kfar Jouz/Kfour (29 March 2022), the same individual was recaptured and photographed the following year (25 May 2023). Two additional records from this locality included the dead adult (7 June 2022) and a live subadult (31 August 2023). Other records include an adult killed by a cat at Deir Al Mokhalles (2 July 2023) and four observations from Marj El Simah in the Rachaya region: a dead adult specimen (29 May 2023), a live smaller adult individual (26 February 2024), exhibiting defensive biting resulting in a mild envenomation case to one of the observers, a juvenile that was killed by locals after entering a house (25 August 2025), and the most recent record of a live adult individual that was active during the morning hours (4 October 2025). The juvenile from Kfar Mechki (14 September 2024; 1,148 m a.s.l.) represents the highest elevation record for Lebanon (record PSL013: the specimen was dead and collected). Additional observations comprise an adult trapped in a greenhouse in Ebba (6 May 2024), a juvenile killed by locals in Bedias (15 July 2024; 17 m a.s.l., the lowest elevation record), and a record from Rihan (15 January 2025; 1,050 m a.s.l.). In total, records comprise 10 adults, three subadults, three juveniles, and one unaged individual, of which 11 were observed alive and six as dead individuals (Table 1).

All documented individuals belonged to the longitudinally striped morph. Metric and meristic data were obtained for four individuals from four localities (see Table 2). Ventrals averaged 166.8 ± 4.7 (160–171) and subcaudals 116.0 ± 2.9 (112–119); dorsal scale rows were constant (17 in all individuals).

**Table 2.** Selected morphometric and meristic data on *Psammophis schokari* from Lebanon.

Field number	Locality	Date	Dorsal Scales	Ventral Scales	Subcaudal Scales	SVL (mm)	HL (mm)	TL (mm)	TotL (mm)
PSL004	Kfar Jouz/Kfour	29 March 2022	17	168	116	-	-	-	-
PSL011	Ebba, Nabatieh	6 May 2024	17	160	112	-	-	-	-
PSL013	Kfar Mechki, Rachaya	14 September 2024	17	168	117	268	13.2	122	390
PSL016	Marj El Simah, Rachaya	4 October 2025	17	171	119	-	-	-	750

The seasonal presence of the 17 records shows that most observations fall within the expected activity period of snakes in the region, from late spring to summer (May–August; *n* = 10). Additional records were obtained in March (*n* = 1), September (*n* = 2), and October (*n* = 1), whereas three observations surprisingly derive from winter months: January (n = 1), February (*n* = 1), and November (*n* = 1).

**Figure 3.**
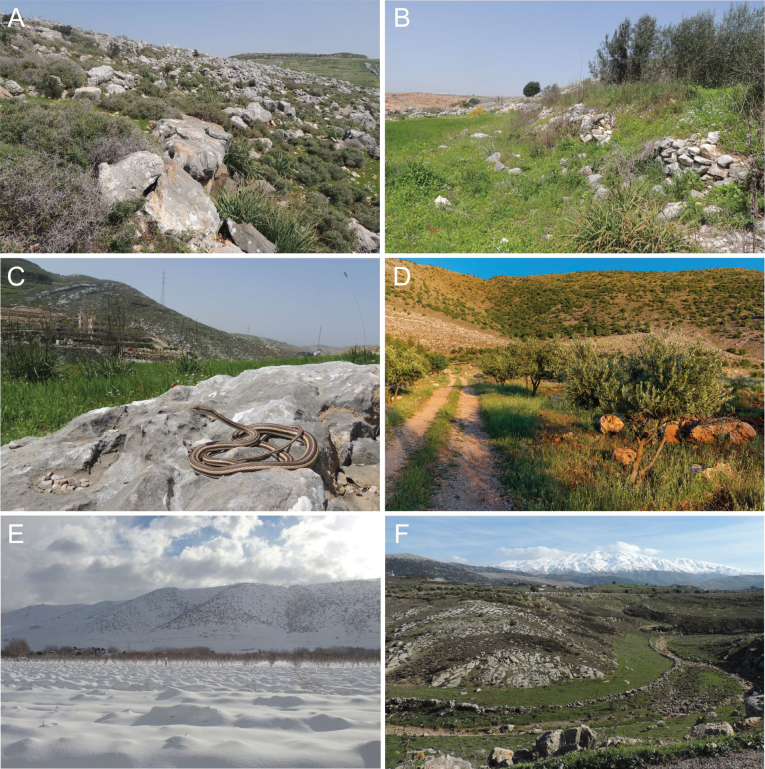
Typical habitats of *Psammophis schokari* in Lebanon, illustrating a mosaic of rocky karstic (carbonate) terrain, grasslands, and agrohabitats. **A**. Rocky karst slope with low-shrub vegetation; **B**. Grassy field margin with stone terraces/low walls; **C**. Individual basking on a large carbonate boulder (all from Kfar Jouz/Kfour; 30 March 2022); **D**. Open olive-grove landscape interspersed with rocky karst (Anjar; 8 May 2022); **E**. The same Anjar area in winter, with seasonal snow cover (10 January 2013); **F**. Karstic plateau with a shallow valley (Marj El Simah; 27 February 2024). Photographs by Rami Khashab, Tsolag Hergelian, and Daniel Jablonski.

Despite generally being considered as species of arid habitats, in Lebanon, it inhabits rocky, shrubby hillsides with poor tree cover as well as open agricultural areas with rocky walls and even dry rocky riversides (Fig. 3). During the winter, few localities with confirmed presence are irregularly covered by snow (Fig. 3E). The species shares the habitat with other reptiles, i.e. *Testudo graeca*, *Pseudopus apodus levantinus*, *Chalcides guentheri*, *Chalcides ocellatus*, *Heremites vittatus*, *Eumeces schneideri*, *Ablepharus rueppellii*, *Laudakia vulgaris*, *Phoenicolacerta laevis*, *Lacerta media*, *Ophisops elegans*, *Ptyodactylus puiseuxi*, *Hemidactylus turcicus*, *Mediodactylus orientalis*, *Xerotyphlops syriacus*, *Letheobia simoni*, *Eryx jaculus*, *Micrelaps muelleri*, *Dolichophis jugularis*, *Hemorrhois nummifer*, *Platyceps collaris*, *Eirenis rothii*, *E. decemlineatus*, *E. lineomaculatus*, *Rhynchocalamus melanocephalus*, *Telescopus fallax*, *Malpolon insignitus*, *Natrix tessellata*, and *Daboia palaestinae*.

## Discussion

More than 160 years after the first doubtful report of the species from Beirut (Jan 1863), we have confirmed the well-established presence of *P. schokari* in Lebanon and, for the first time, investigated its basic genetic affiliation. This represents an important step toward understanding the species’ occurrence and ecological adaptability at the northern edge of its range, where Lebanon lies. As noted earlier, the presence of *P. schokari* in the country was either uncertain (Hraoui-Bloquet 1981) or explicitly questioned, partly due to the possibility of imports from Egypt for the pet trade (Werner 1939). However, our data clearly confirm that the species has an extensive distribution across the southern part of Lebanon, linked to populations from the south-western parts of the species range (Figs 1, 2 and Aidek et al. 2023).

Our results fit into the broader phylogeographic framework outlined by Gonçalves et al. (2018), who identified six major mitochondrial lineages of *P. schokari* across North Africa and Western Asia. Unexpectedly, the genetic relationships recovered here are not geographically straightforward. Although the Lebanese samples belong to the “Middle Eastern lineage”, the two haplotypes detected in southern Lebanon (sampled ~5 km apart) form a tight local cluster (separated by a single mutational step) yet show closer affinity to northeastern African/Sinai haplotypes than to those currently documented from the southern Levant. This pattern is consistent with a scenario of historical range expansion from North Africa into the Levant via the Sinai corridor, followed by local persistence and differentiation at the northern range margin. In Lebanon, the Litani–Hasbani drainage may have functioned as a biogeographic filter shaping local structure, while the associated river valleys could also have acted as dispersal corridors facilitating movements within southern Lebanon.

From a temporal perspective, divergence among major *P. schokari* lineages was placed in the Pliocene, with subsequent structuring during the Pleistocene (Gonçalves et al. 2018). The Lebanese haplotypes may therefore represent signatures of late Pleistocene expansions, preserved in peripheral populations at the northern edge of the species’ range. This interpretation is consistent with recent evidence for other African-derived lineages in southern Lebanon (e.g. *Letheobia simoni*; see discussion by Jablonski et al. 2024), suggesting that the region may have repeatedly received colonizers from Africa through distinct historical dispersal events. Gonçalves et al. (2018) further emphasized the role of climatic oscillations and ecological connectivity between North Africa and the Levant in shaping diversification and distribution patterns in *P. schokari*. Accordingly, the affinity of Lebanese haplotypes to Egypt/Sinai is compatible with historical colonization followed by persistence in local refugia during unfavourable climatic periods. However, large parts of the species’ range remain unsampled, and the inferred phylogeographic structure may change with denser geographic coverage.

Regarding regional connectivity, although the southern Levant currently harbours different mitochondrial haplotypes (Fig. 1), we cannot exclude that these haplotypes also occur in southern Lebanon but remain undetected due to sparse sampling (e.g. localities 7, 10, 13, 15; Fig. 2). Broader genetic sampling, particularly along putative dispersal axes such as the Litani and Hasbani River corridors (Fig. 2), will be necessary to test this prediction and to clarify whether the observed pattern reflects true historical colonization dynamics, secondary contact, or gaps in sampling.

A potential historical introduction, as suggested by Werner (1939), may explain the record from Beirut (Jan 1863), since this locality lies far north of the populations discovered here, and no data confirm its current presence despite extensive fieldwork around the capital. Beirut is known for the snake pet trade (e.g. *Natrix tessellata* from Syria; Khashab pers. obs.), although direct evidence for *Psammophis* in this context is lacking. Alternatively, if a population had indeed existed in Beirut due to past natural dispersions, it may have become extinct due to intense urban expansion and overpopulation in and around the city, which have led to the disappearance of suitable habitats in western and southwestern Beirut over recent decades. In contrast, the second historical record from Sidon falls within the currently known range of the species (see localities 8 and 17 in Fig. 2).

*Psammophis schokari* is a species with high habitat tolerance, and our data from Lebanon highlight important ecological particularities at the northern edge of its distribution. Contrary to Werner’s (1939) and subsequent assumptions that the species is strictly desert-dwelling, our observations document its ability to occupy rocky hillsides with Mediterranean vegetation and even agricultural landscapes, consistent with reports from other parts of its range (e.g. Amr and Disi 2011; Bar et al. 2021). As suggested by a few studies, the colour polymorphism (light snakes without stripes are rather present in deserts) may be an adaptation to habitats that allow better survival (Lahav and Dmi’El 1996; Kark et al. 1997). In Lebanon, the species appears to be represented exclusively by the longitudinally striped morph, which may be adaptive in the rocky and grassy habitats it occupies. The limited morphological data we collected (Table 2) fall within the range previously reported from the region, although subcaudal counts are slightly higher (cf. Kark et al. 1997). The species also occurs at elevations exceeding 1,100 m a.s.l. (and up to 3,500 m in Iran; Kamali 2020) and was repeatedly recorded during the coldest months (January, February, and November; Table 1), when reptile surface activity is typically strongly reduced. These winter records likely reflect opportunistic activity during favourable microclimatic windows and the buffering effects of relatively mild lowland habitats, consistent with its thermophilic ecology. Such ecological flexibility aligns with the broad niche breadth and apparent capacity to persist under fluctuating climatic regimes inferred for *P. schokari* in North Africa (Gonçalves et al. 2018), indicating that the species is not restricted to deserts but can occupy a wide spectrum of habitats, including areas that are seasonally snow-covered (Fig. 3).

This ability to exploit heterogeneous environments suggests that *P. schokari* is less constrained by seasonality than many other snakes in the Levant and Western Asia, which may help explain its capacity to disperse and establish populations in biogeographically complex regions across wide areas of the central and southern Western Palearctic, including mountainous Lebanon. Our results also highlight the Levant as an important biogeographic bridge connecting African and Asian faunas and underscore the need for further integrative research to reconstruct the colonization routes and adaptive history of desert reptiles at the margins of their ranges.

## Additional information

### Use of AI

No use of AI was reported.

### Author ORCIDs

Daniel Jablonski https://orcid.org/0000-0002-5394-0114

Rami Khashab https://orcid.org/0009-0006-9608-6014

Riyad A. Sadek https://orcid.org/0009-0005-8392-5942

### Data availability

All of the data that support the findings of this study are available in the main text or Supplementary Information.

## Supplementary materials

Supplementary material 1 Original photo vouchers of recorded individuals Data type zip Explanation note Confirmatory photographs of the recorded Psammophis schokari specimens from Lebanon (labelled PSL001–PSL016), corresponding to Table 1 in the main article. https://binary.pensoft.net/file/1524026This dataset is made available under the Open Database License (http://opendatacommons.org/licenses/odbl/1.0/). The Open Database License (ODbL) is a license agreement intended to allow users to freely share, modify, and use this Dataset while maintaining this same freedom for others, provided that the original source and author(s) are credited. Daniel Jablonski, Rami Khashab, Riyad A. Sadek
