# Supplementary figures and images for: From yellow deserts to white mountains: confirmed occurrence and genetic affiliation of Psammophis schokari (Forskål, 1775) (Serpentes, Psammophiidae) in Lebanon
Source: Zookeys. 2026 Feb 2;1268:1–12. doi: 10.3897/zookeys.1268.177920 (PMC12887587; doi:10.3897/zookeys.1268.177920)

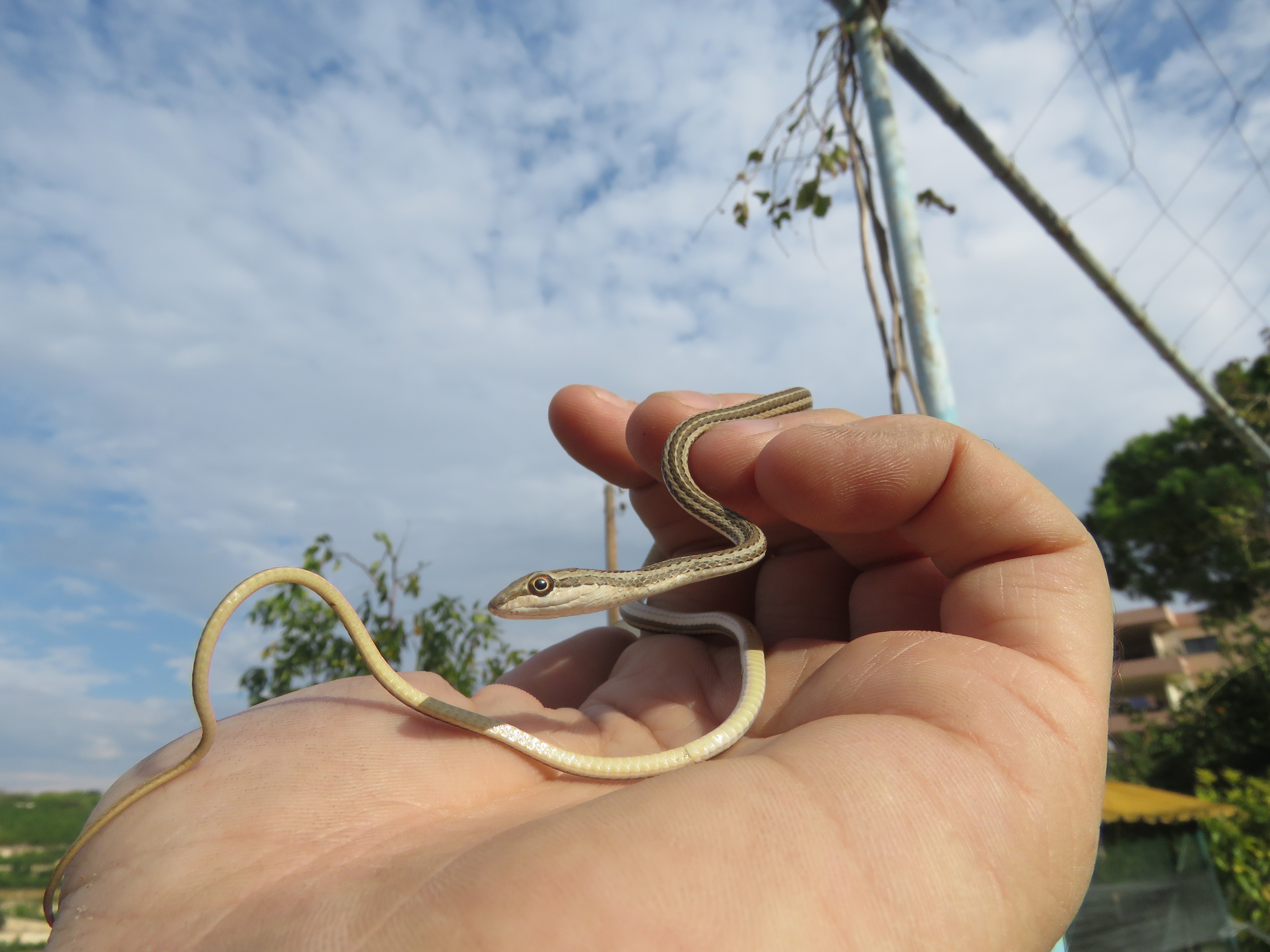

Supplement: Supplementary material 1 — Original photo vouchers of recorded individuals [file zookeys-1268-001_article-177920__-s001.zip › Supplementary_data/PSL001 (1).JPG]

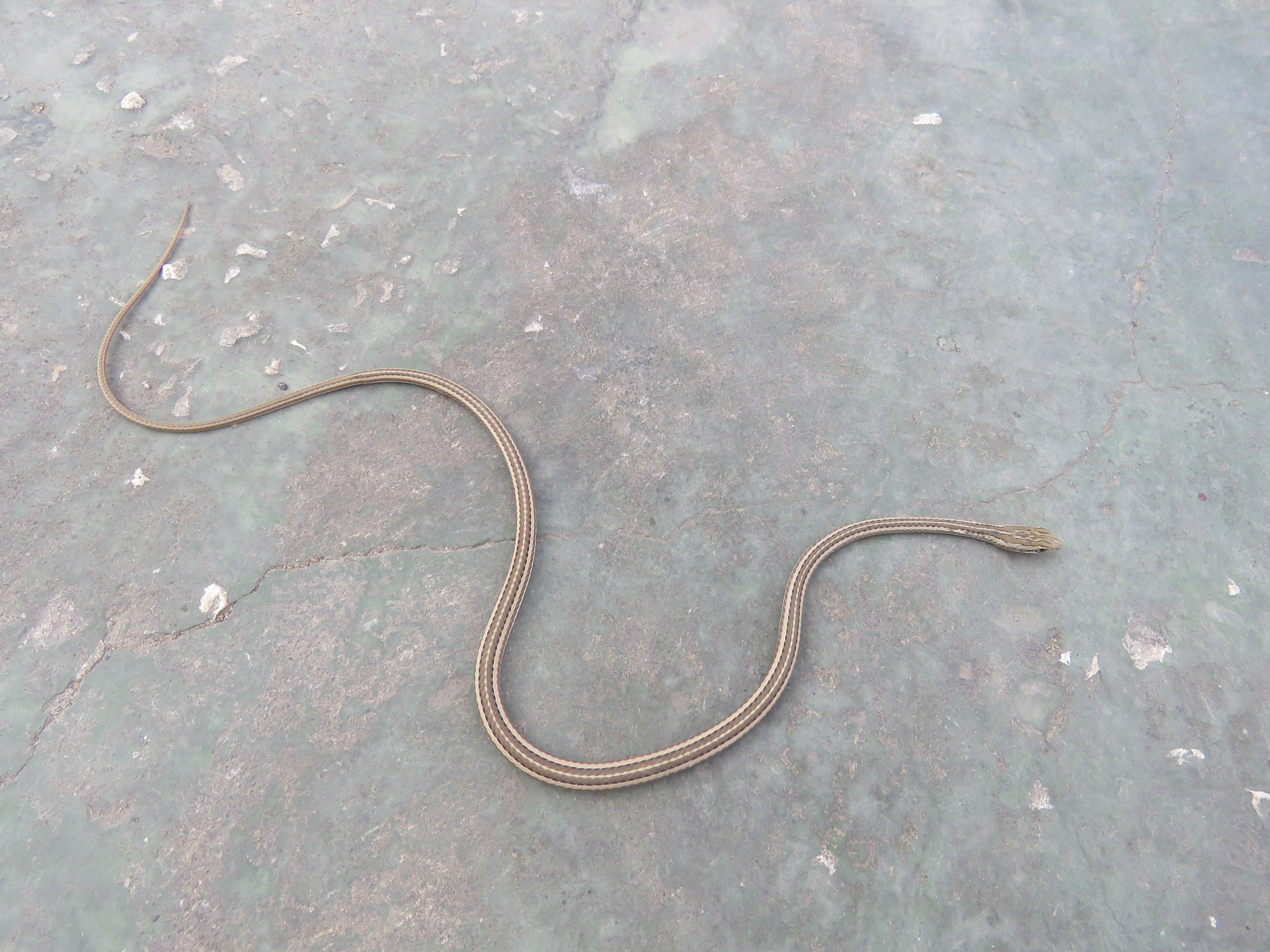

Supplement: Supplementary material 1 — Original photo vouchers of recorded individuals [file zookeys-1268-001_article-177920__-s001.zip › Supplementary_data/PSL001 (2).JPG]

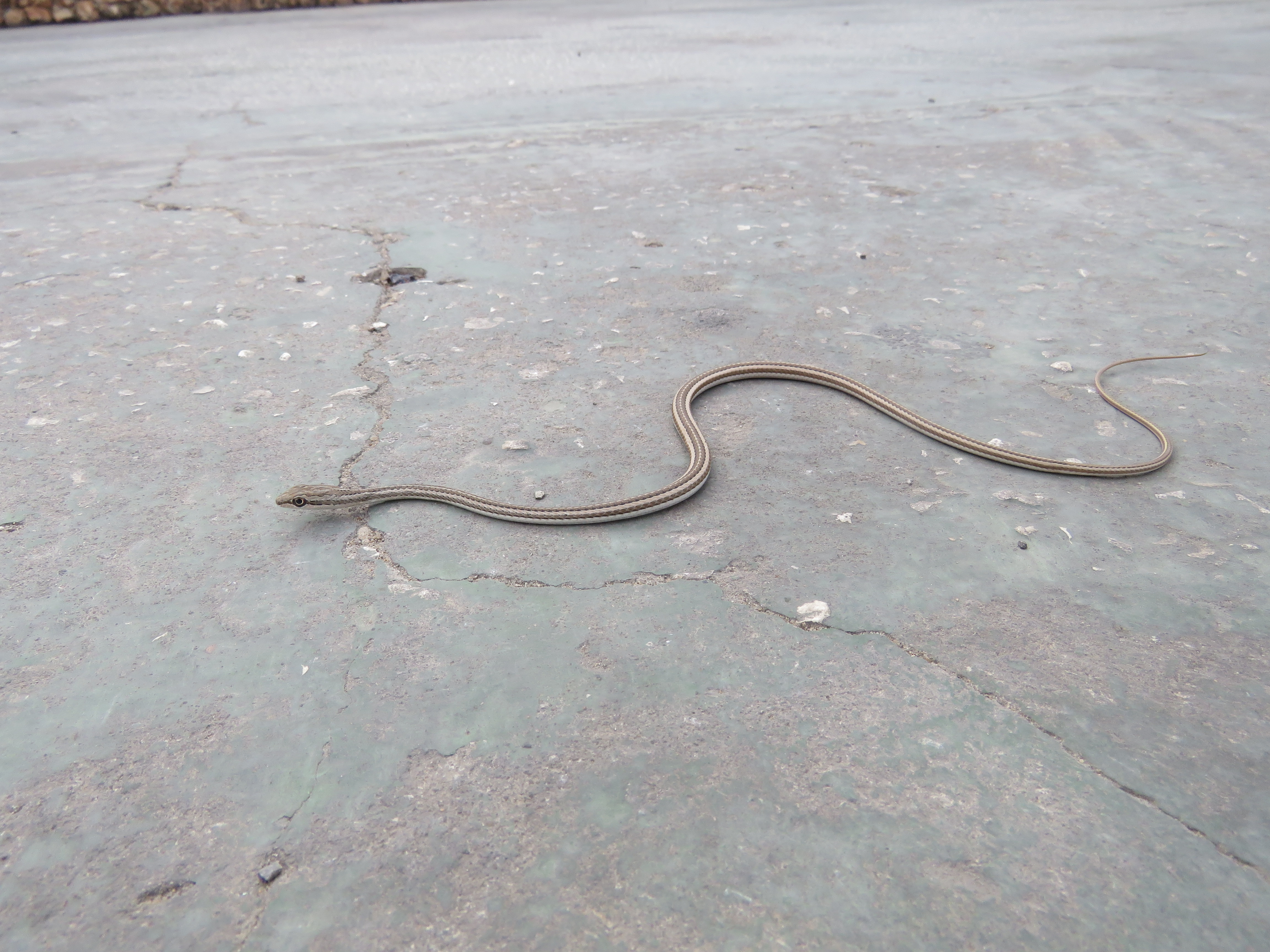

Supplement: Supplementary material 1 — Original photo vouchers of recorded individuals [file zookeys-1268-001_article-177920__-s001.zip › Supplementary_data/PSL001 (3).JPG]

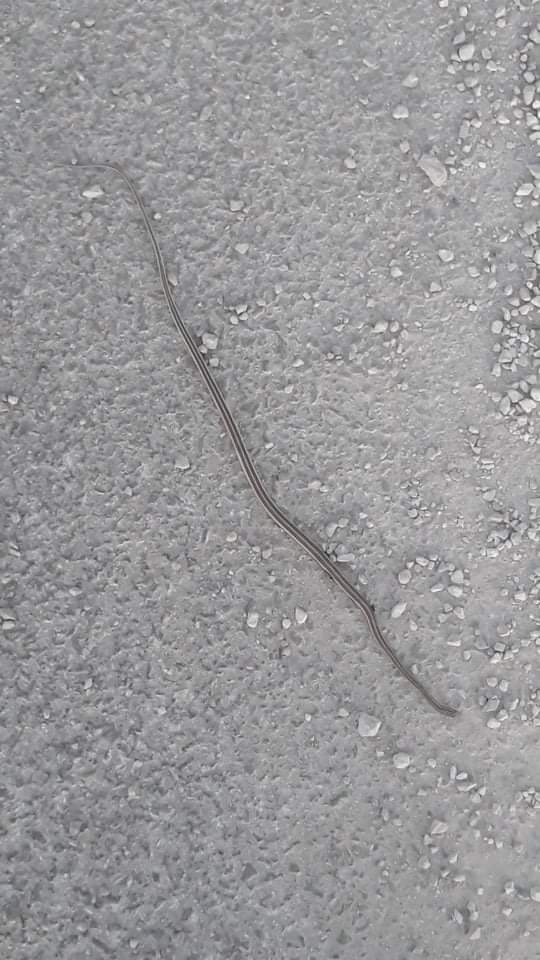

Supplement: Supplementary material 1 — Original photo vouchers of recorded individuals [file zookeys-1268-001_article-177920__-s001.zip › Supplementary_data/PSL003.jpeg]

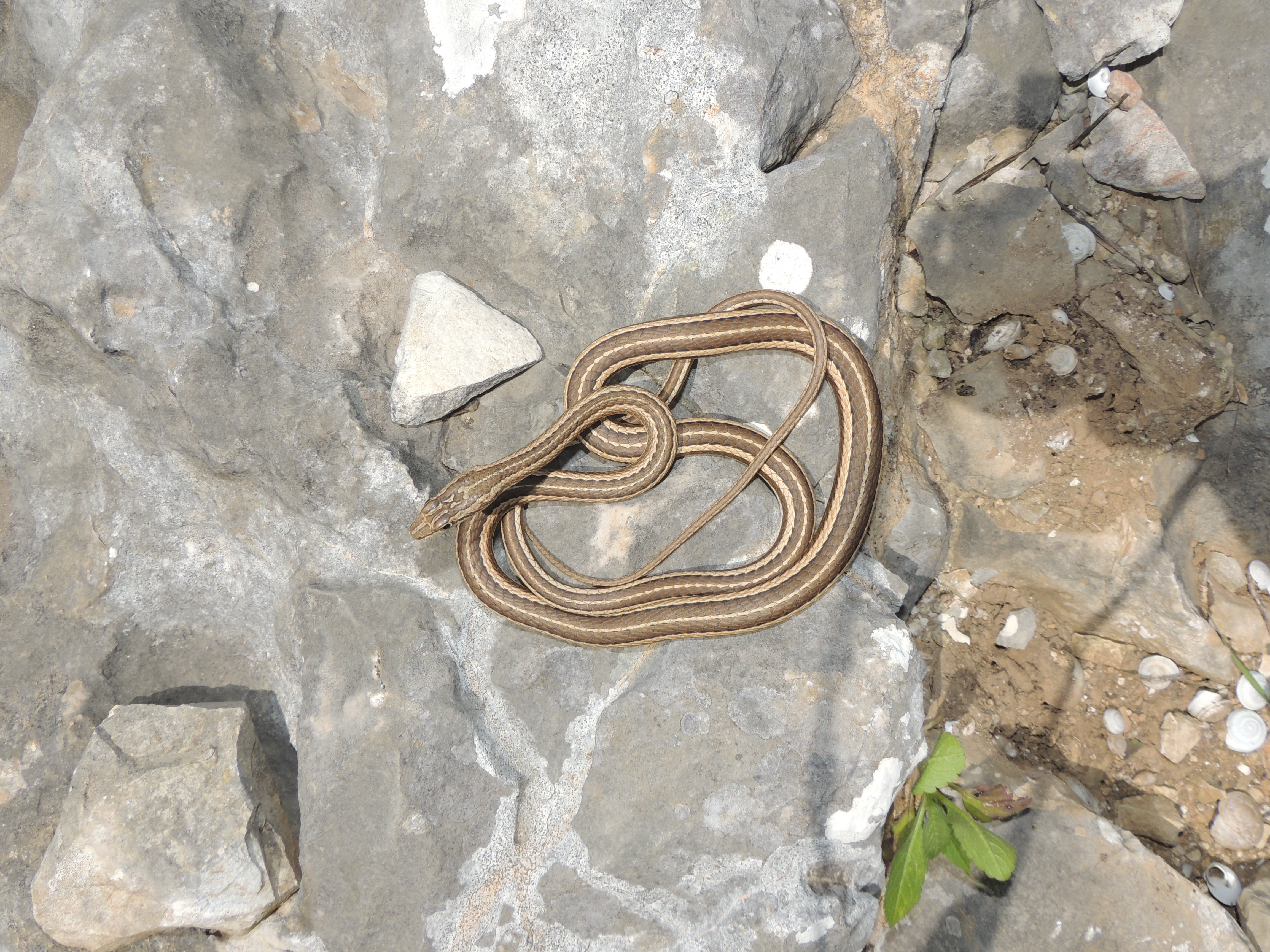

Supplement: Supplementary material 1 — Original photo vouchers of recorded individuals [file zookeys-1268-001_article-177920__-s001.zip › Supplementary_data/PSL004 (1).JPG]

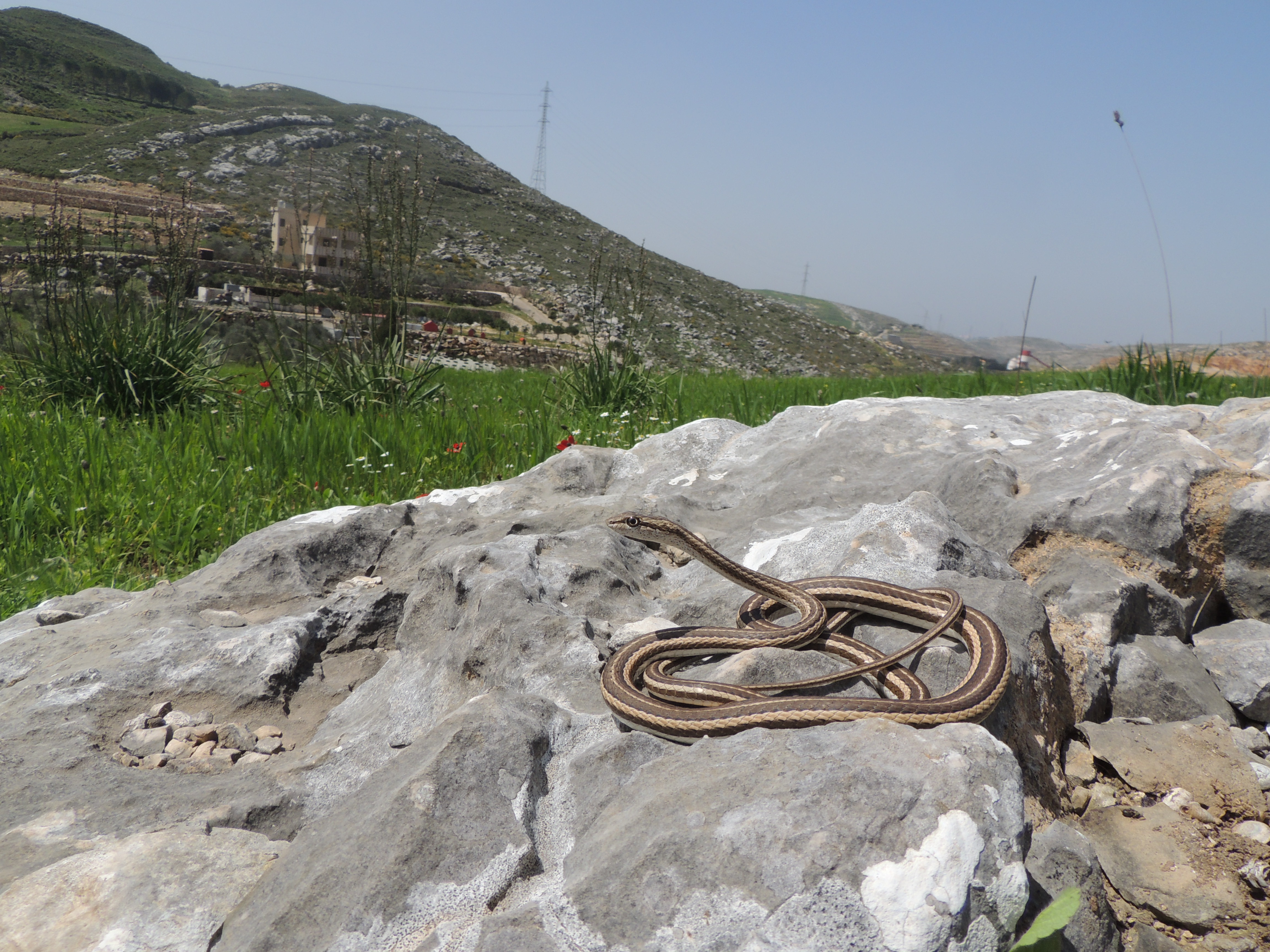

Supplement: Supplementary material 1 — Original photo vouchers of recorded individuals [file zookeys-1268-001_article-177920__-s001.zip › Supplementary_data/PSL004 (2).JPG]

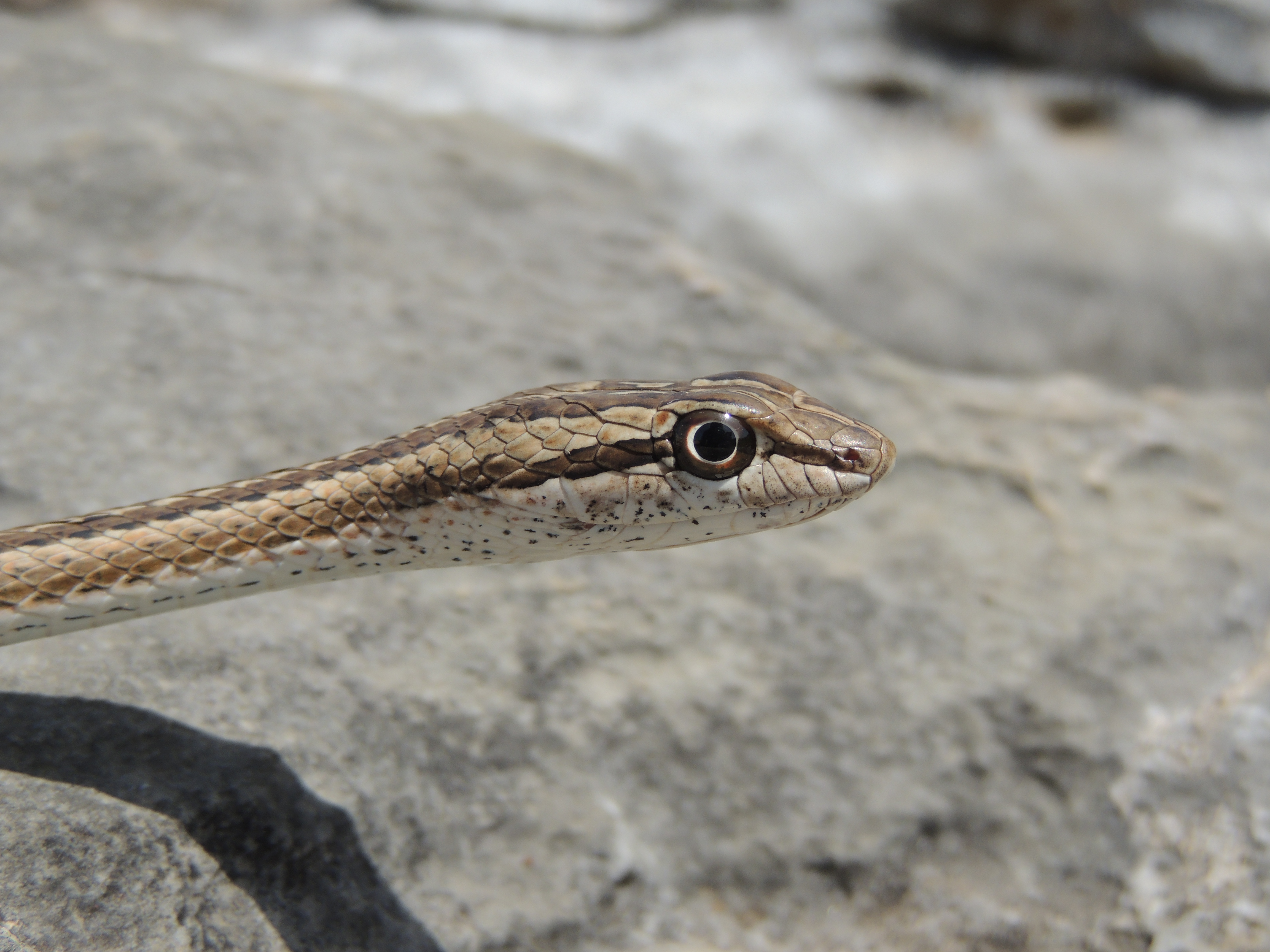

Supplement: Supplementary material 1 — Original photo vouchers of recorded individuals [file zookeys-1268-001_article-177920__-s001.zip › Supplementary_data/PSL004 (3).JPG]

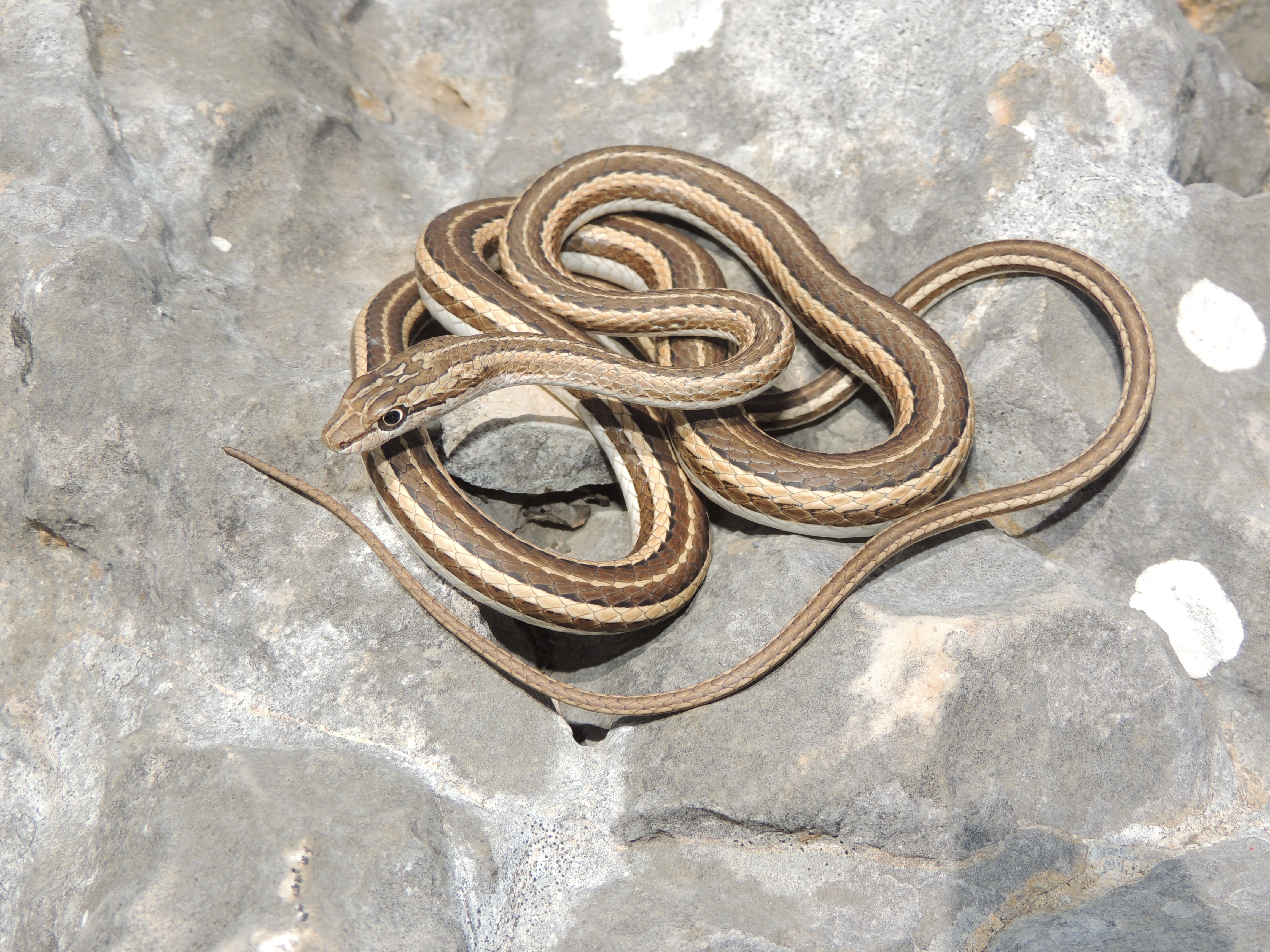

Supplement: Supplementary material 1 — Original photo vouchers of recorded individuals [file zookeys-1268-001_article-177920__-s001.zip › Supplementary_data/PSL004 (4).JPG]

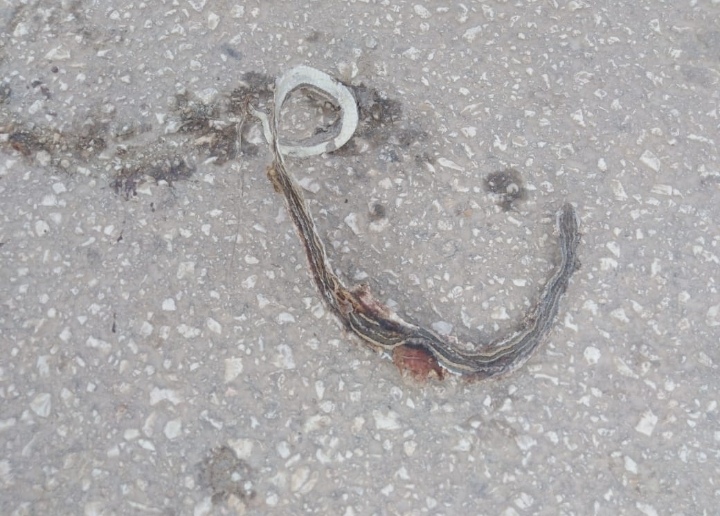

Supplement: Supplementary material 1 — Original photo vouchers of recorded individuals [file zookeys-1268-001_article-177920__-s001.zip › Supplementary_data/PSL005.jpg]

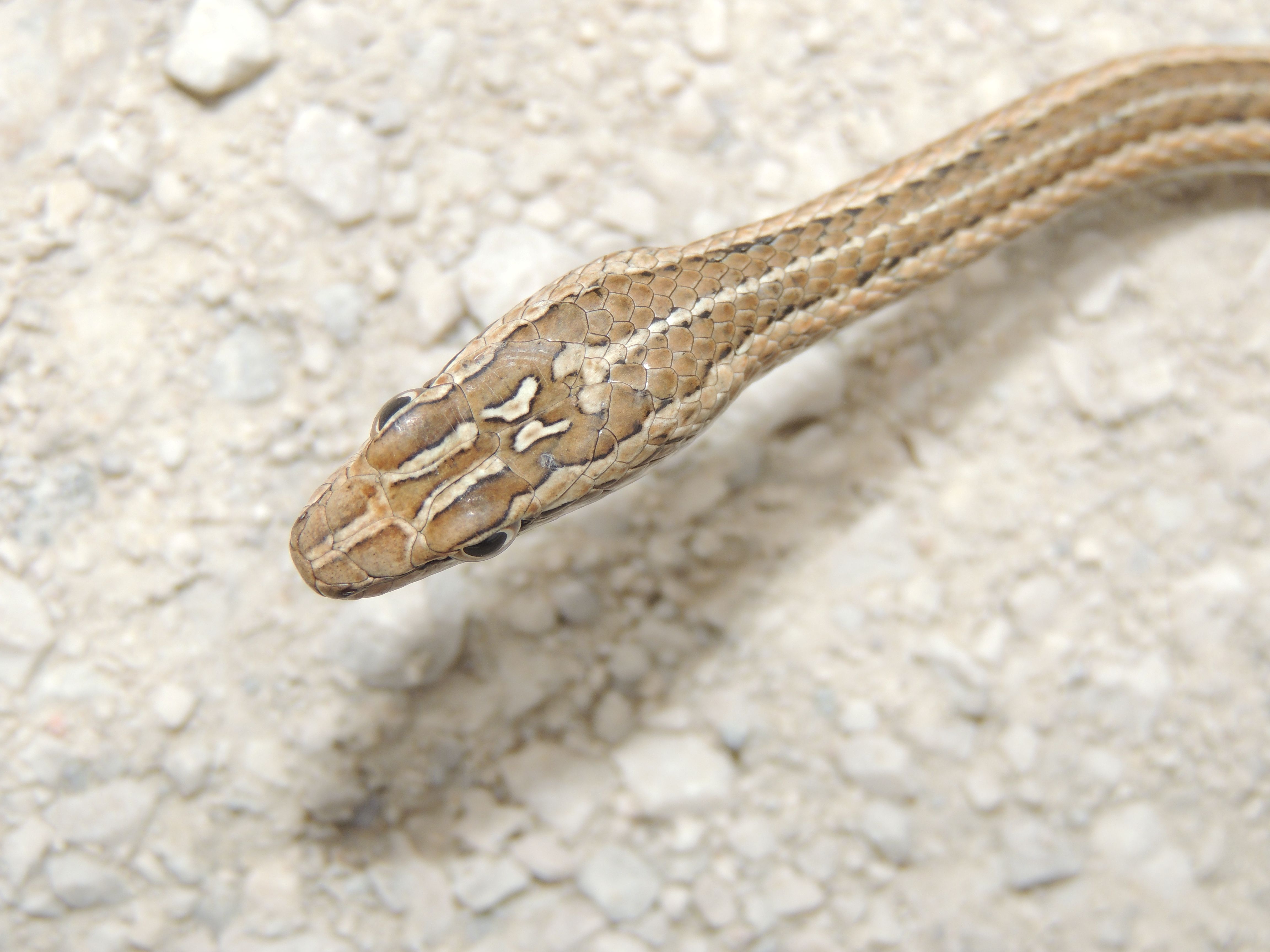

Supplement: Supplementary material 1 — Original photo vouchers of recorded individuals [file zookeys-1268-001_article-177920__-s001.zip › Supplementary_data/PSL006 (1).JPG]

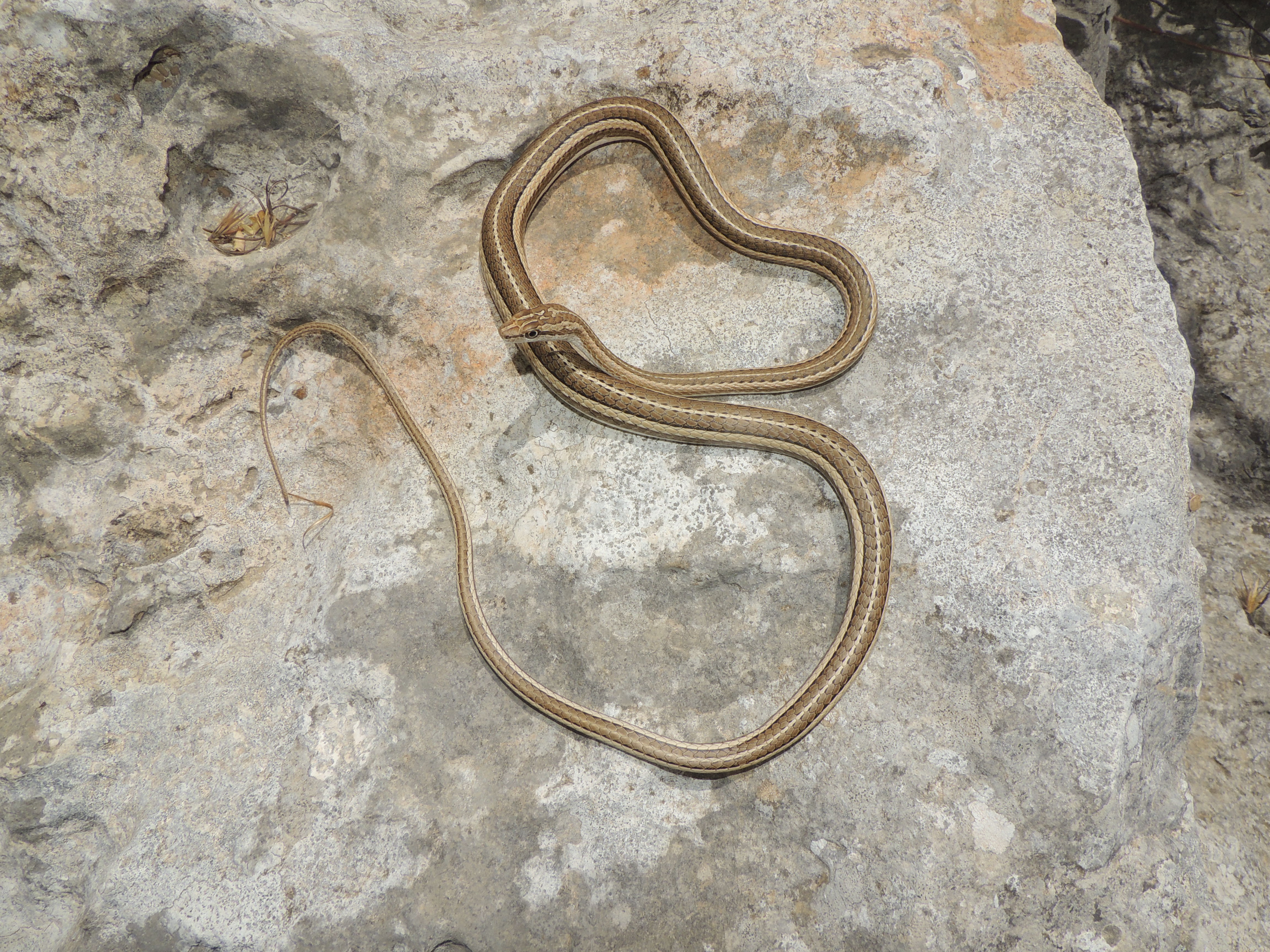

Supplement: Supplementary material 1 — Original photo vouchers of recorded individuals [file zookeys-1268-001_article-177920__-s001.zip › Supplementary_data/PSL006 (2).JPG]

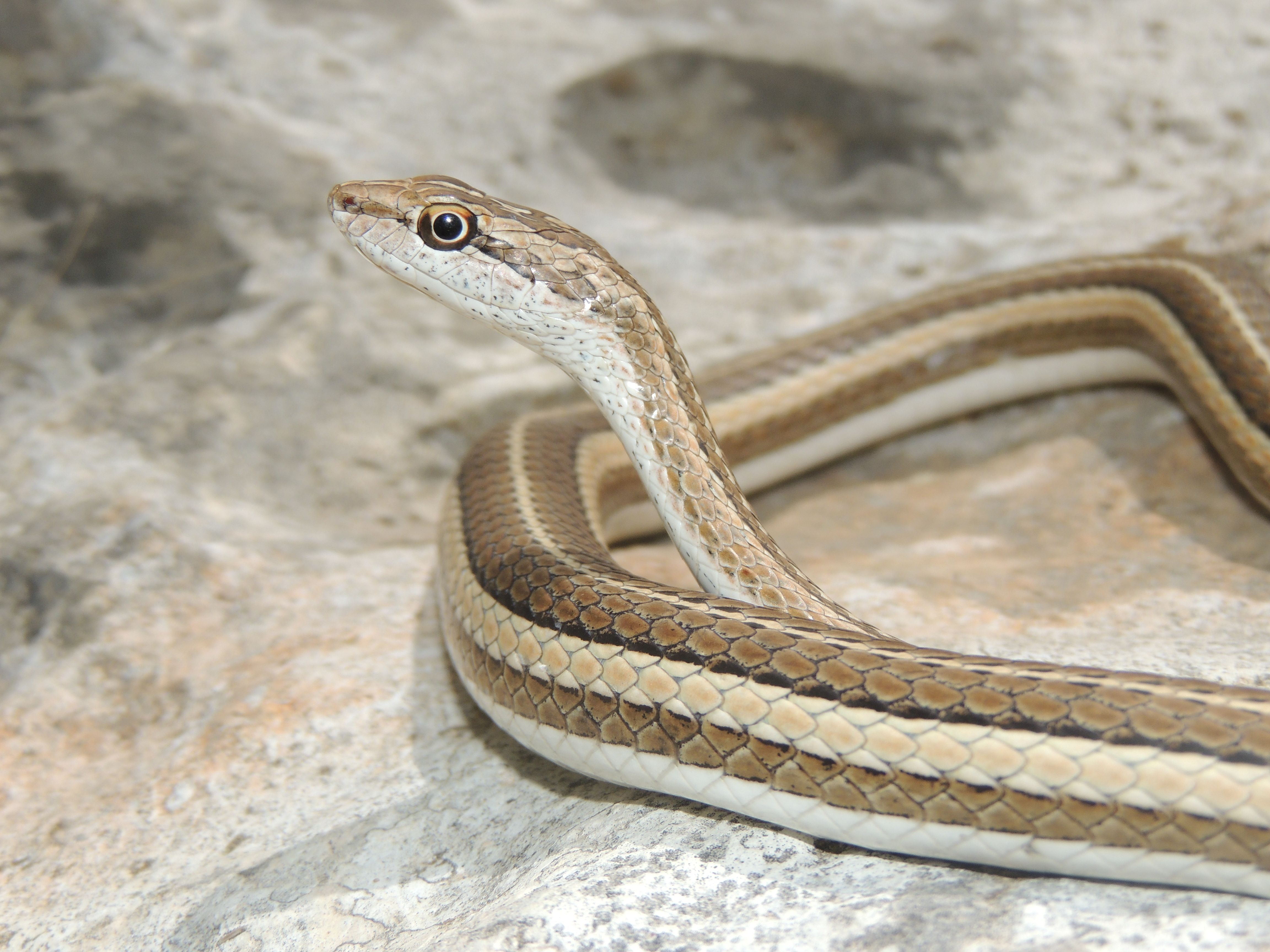

Supplement: Supplementary material 1 — Original photo vouchers of recorded individuals [file zookeys-1268-001_article-177920__-s001.zip › Supplementary_data/PSL006 (3).JPG]

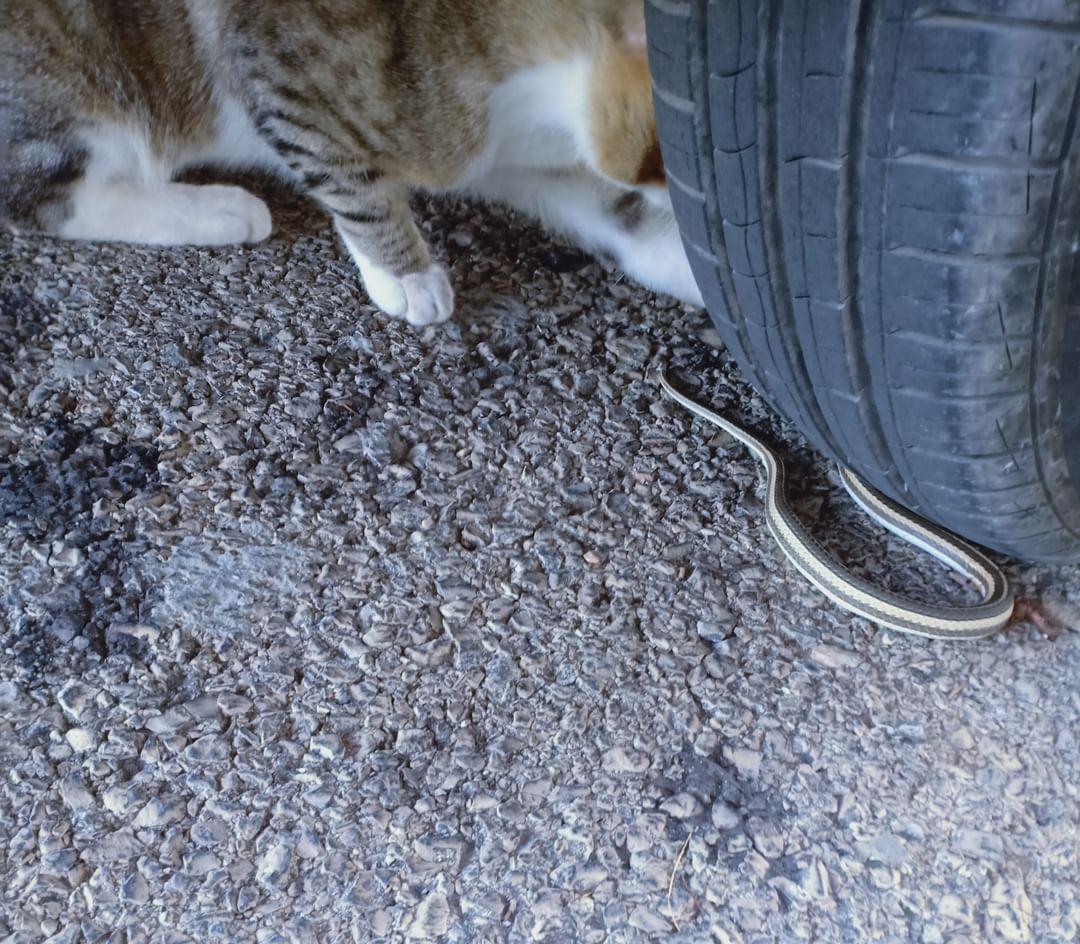

Supplement: Supplementary material 1 — Original photo vouchers of recorded individuals [file zookeys-1268-001_article-177920__-s001.zip › Supplementary_data/PSL008.jpg]

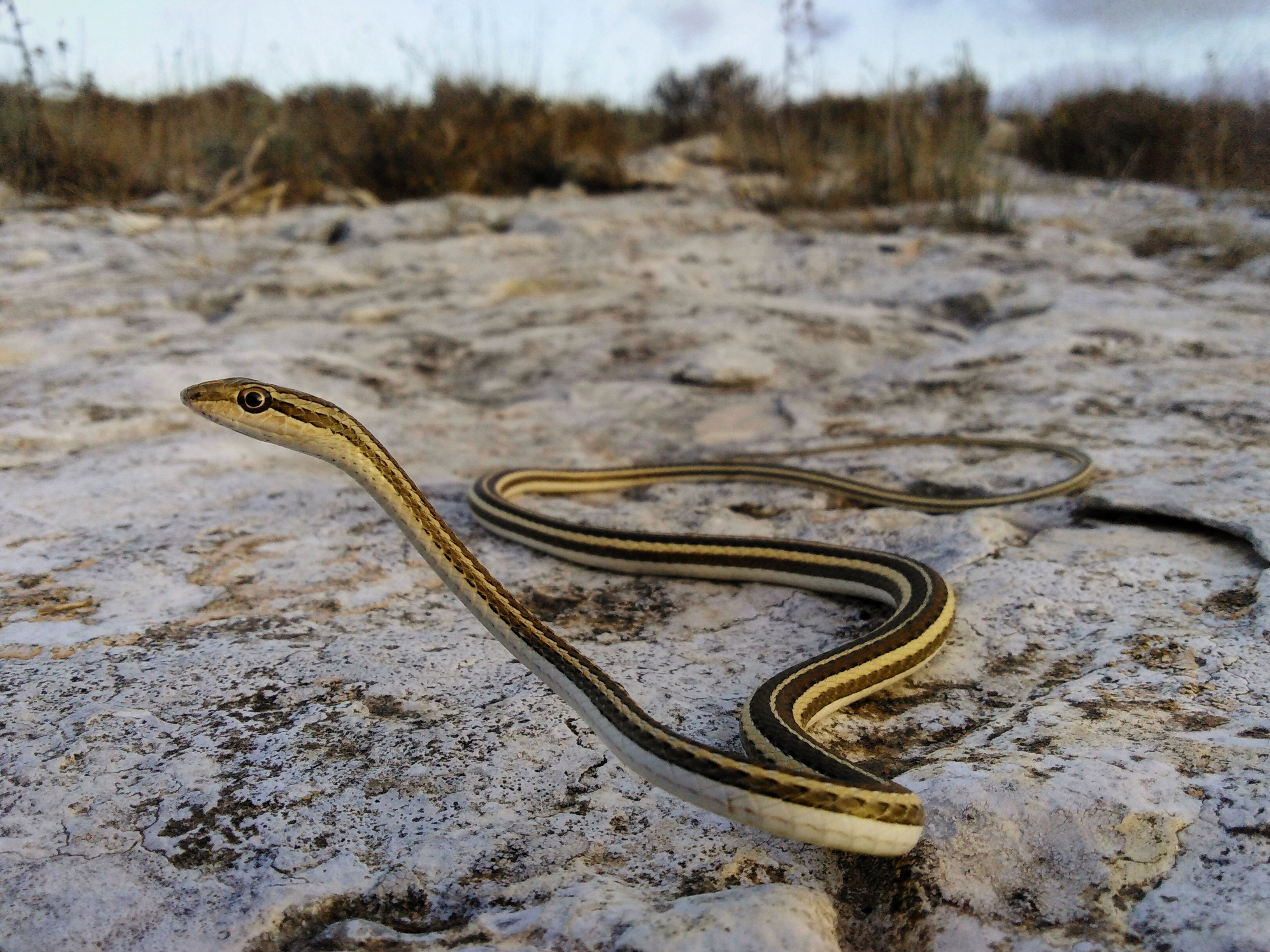

Supplement: Supplementary material 1 — Original photo vouchers of recorded individuals [file zookeys-1268-001_article-177920__-s001.zip › Supplementary_data/PSL009.jpg]

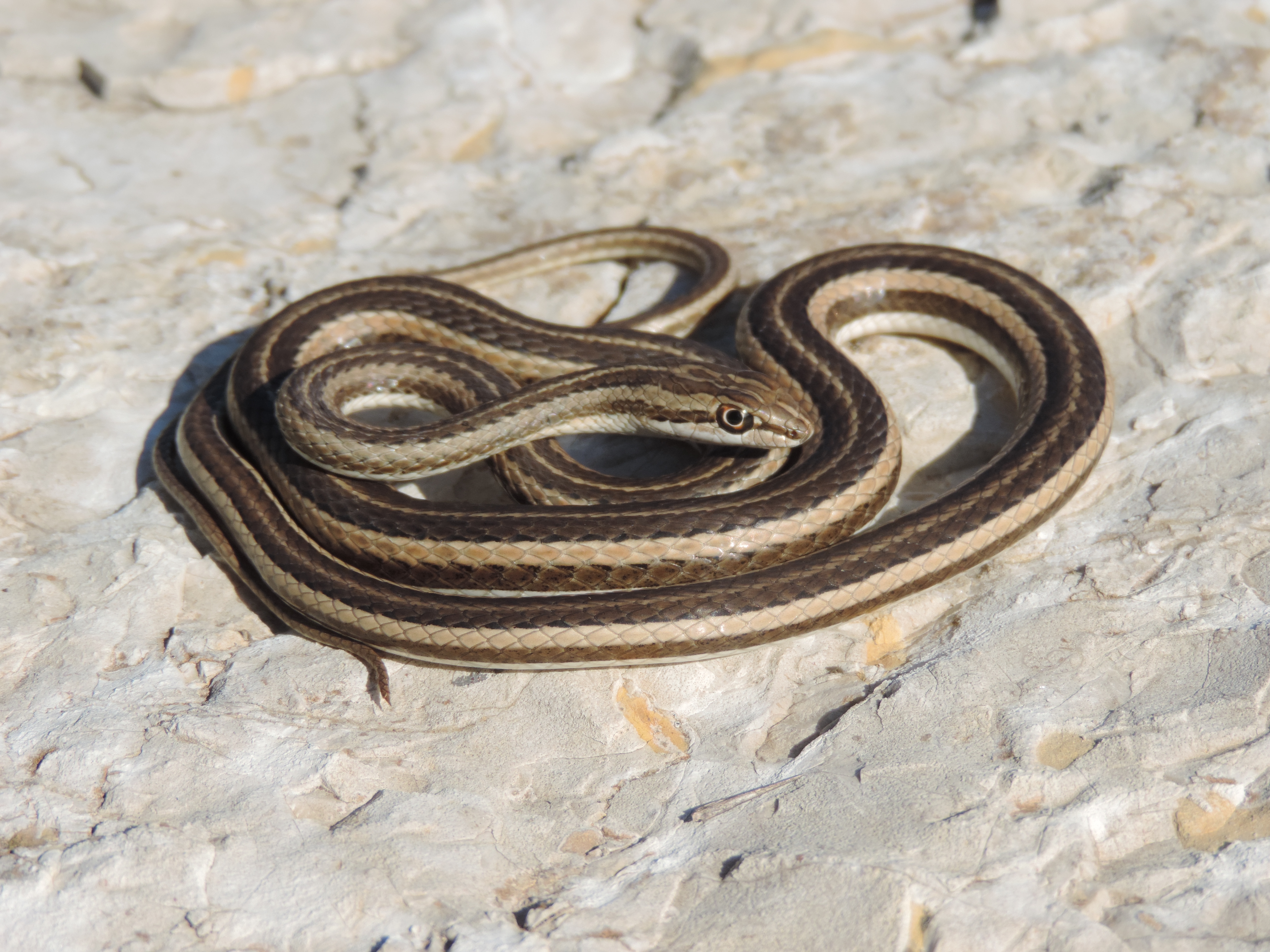

Supplement: Supplementary material 1 — Original photo vouchers of recorded individuals [file zookeys-1268-001_article-177920__-s001.zip › Supplementary_data/PSL010 (1).JPG]

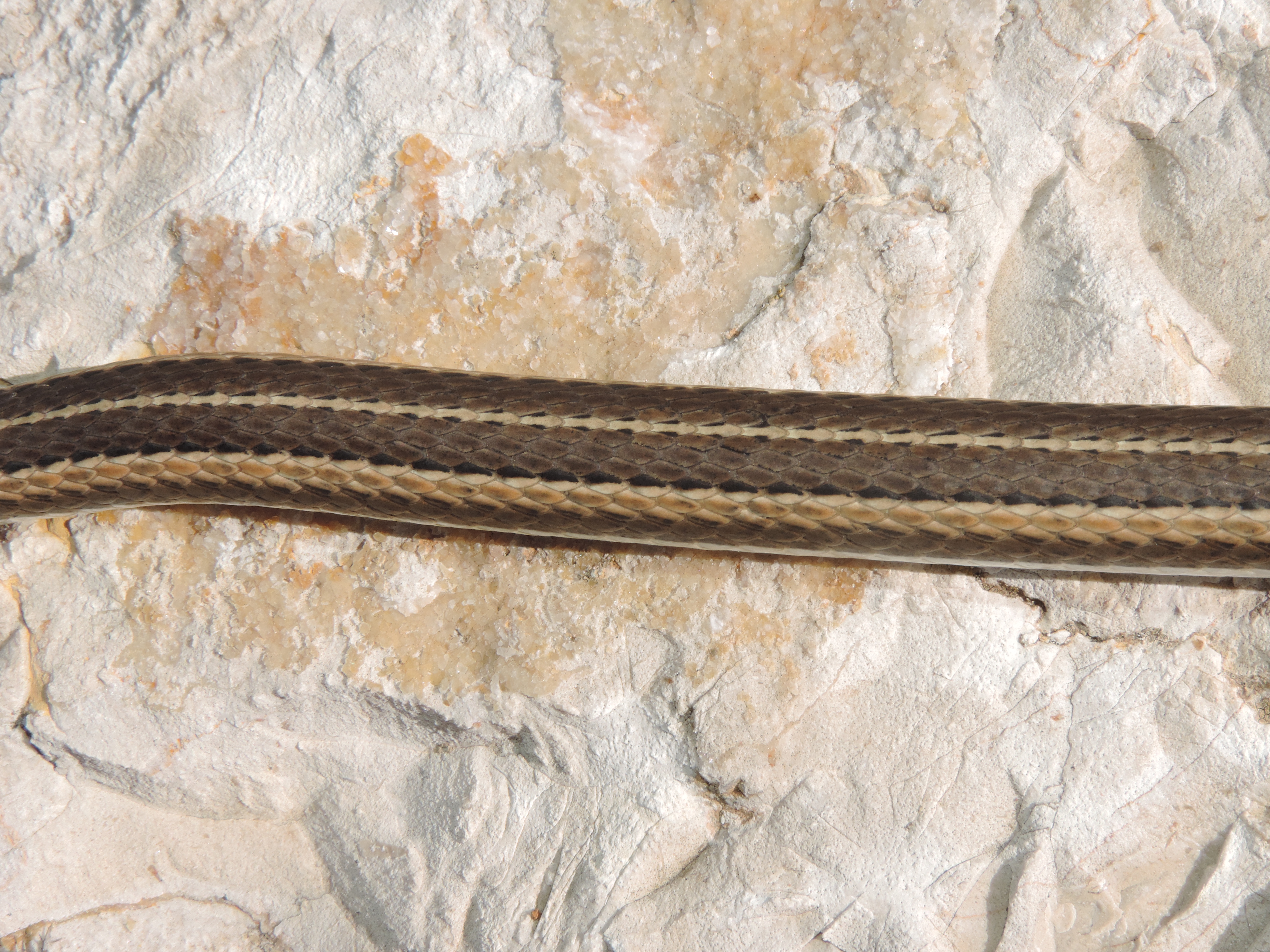

Supplement: Supplementary material 1 — Original photo vouchers of recorded individuals [file zookeys-1268-001_article-177920__-s001.zip › Supplementary_data/PSL010 (2).JPG]

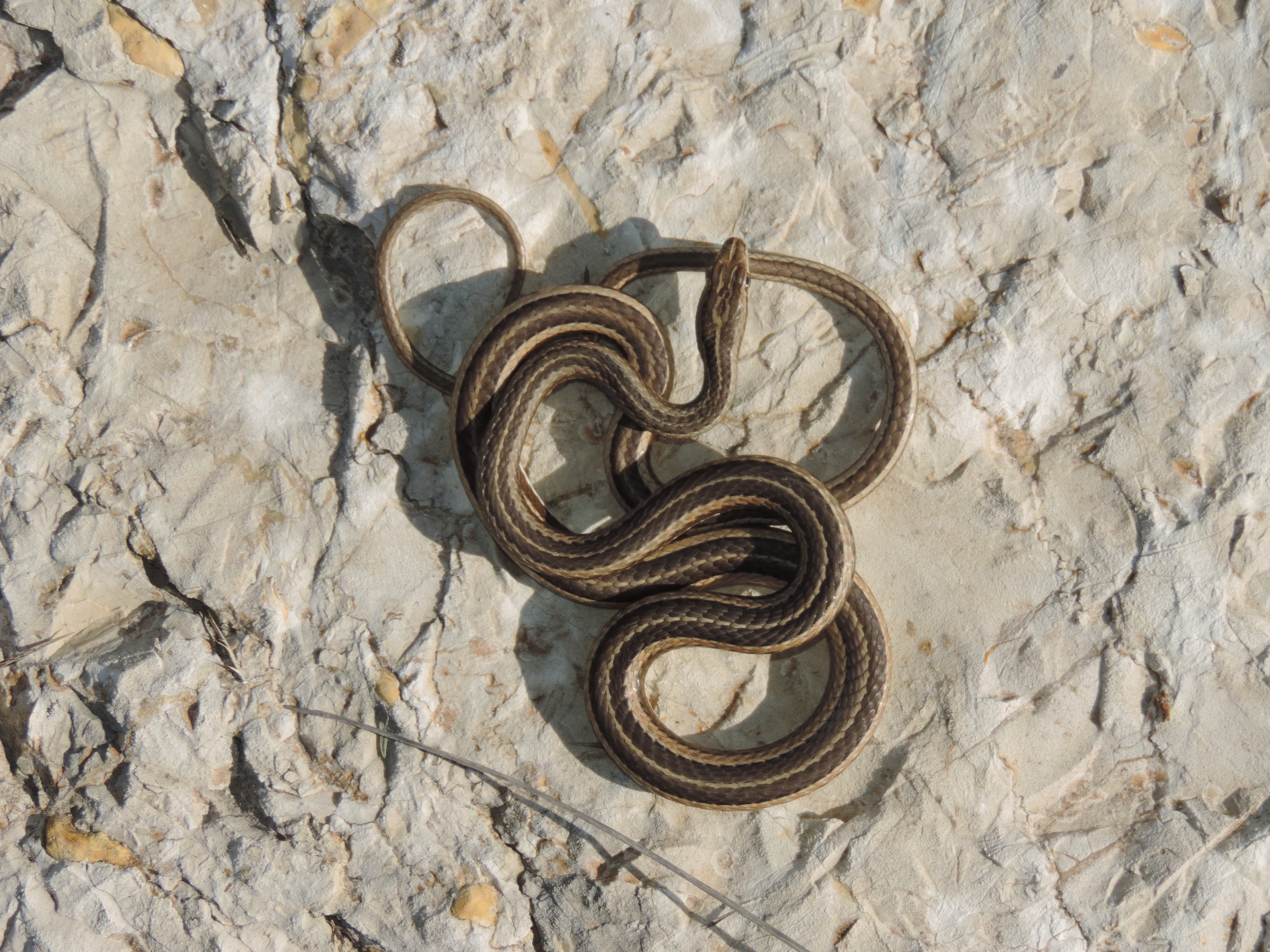

Supplement: Supplementary material 1 — Original photo vouchers of recorded individuals [file zookeys-1268-001_article-177920__-s001.zip › Supplementary_data/PSL010 (3).JPG]

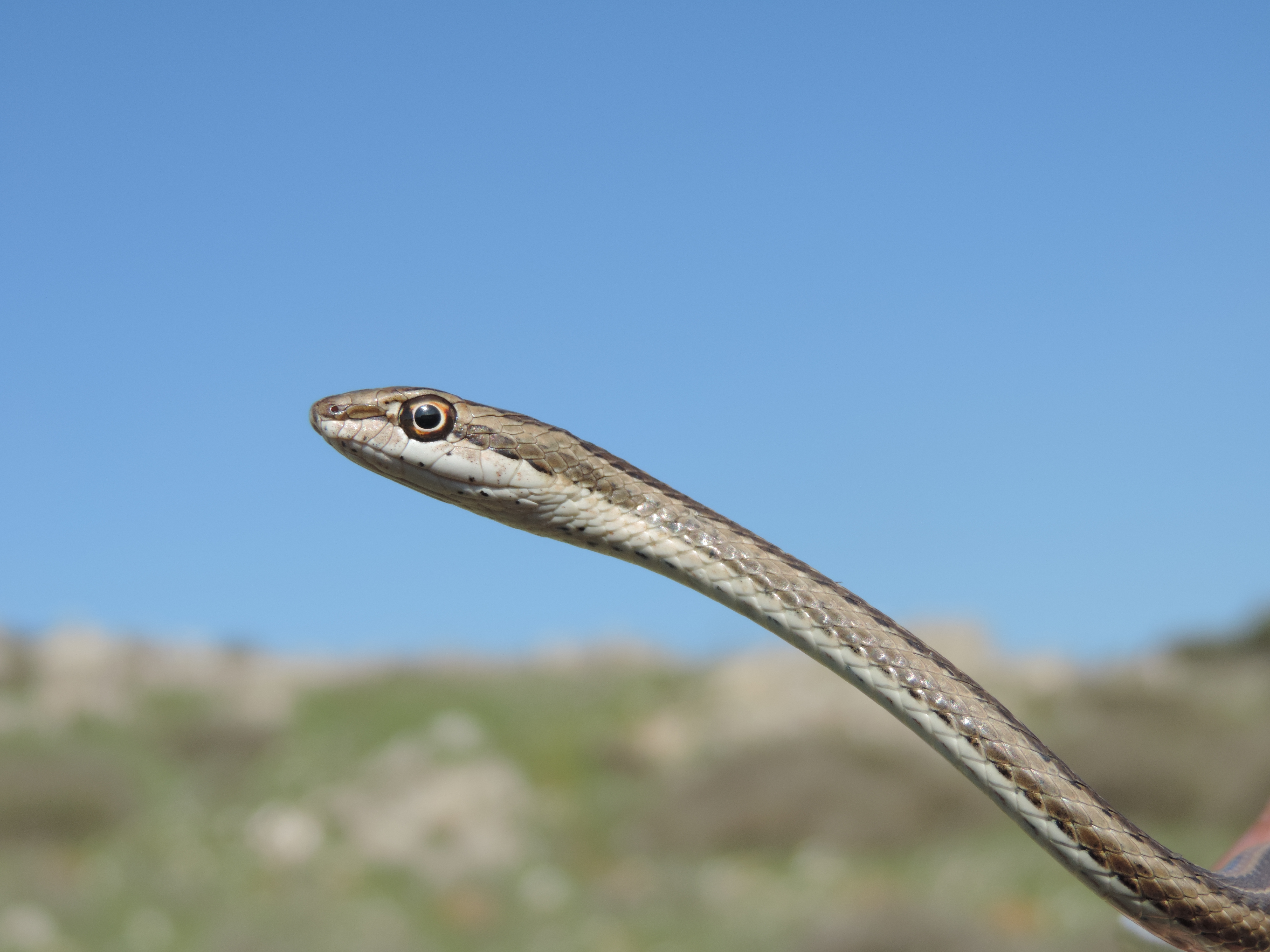

Supplement: Supplementary material 1 — Original photo vouchers of recorded individuals [file zookeys-1268-001_article-177920__-s001.zip › Supplementary_data/PSL010 (4).JPG]

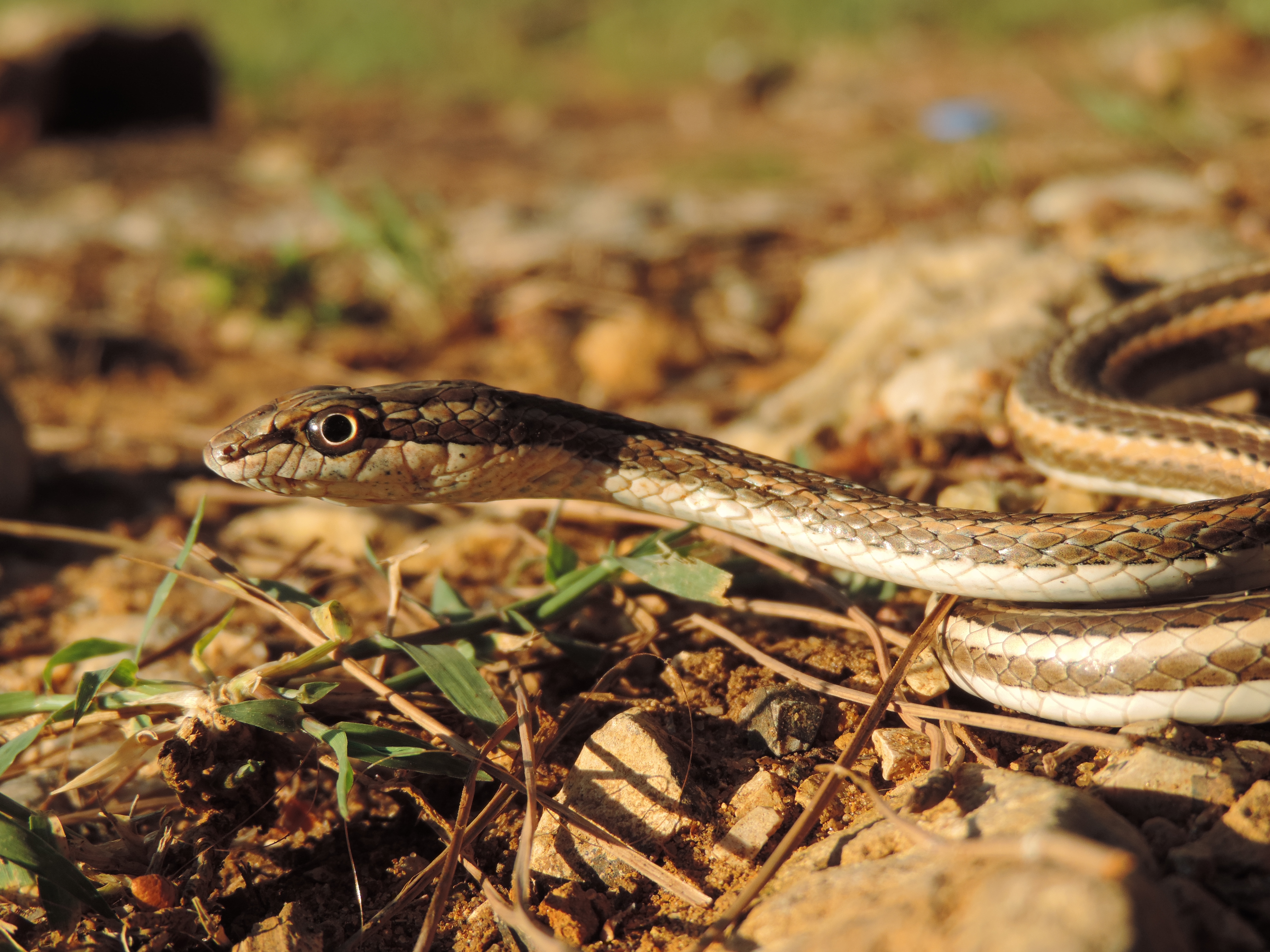

Supplement: Supplementary material 1 — Original photo vouchers of recorded individuals [file zookeys-1268-001_article-177920__-s001.zip › Supplementary_data/PSL011 (1).JPG]

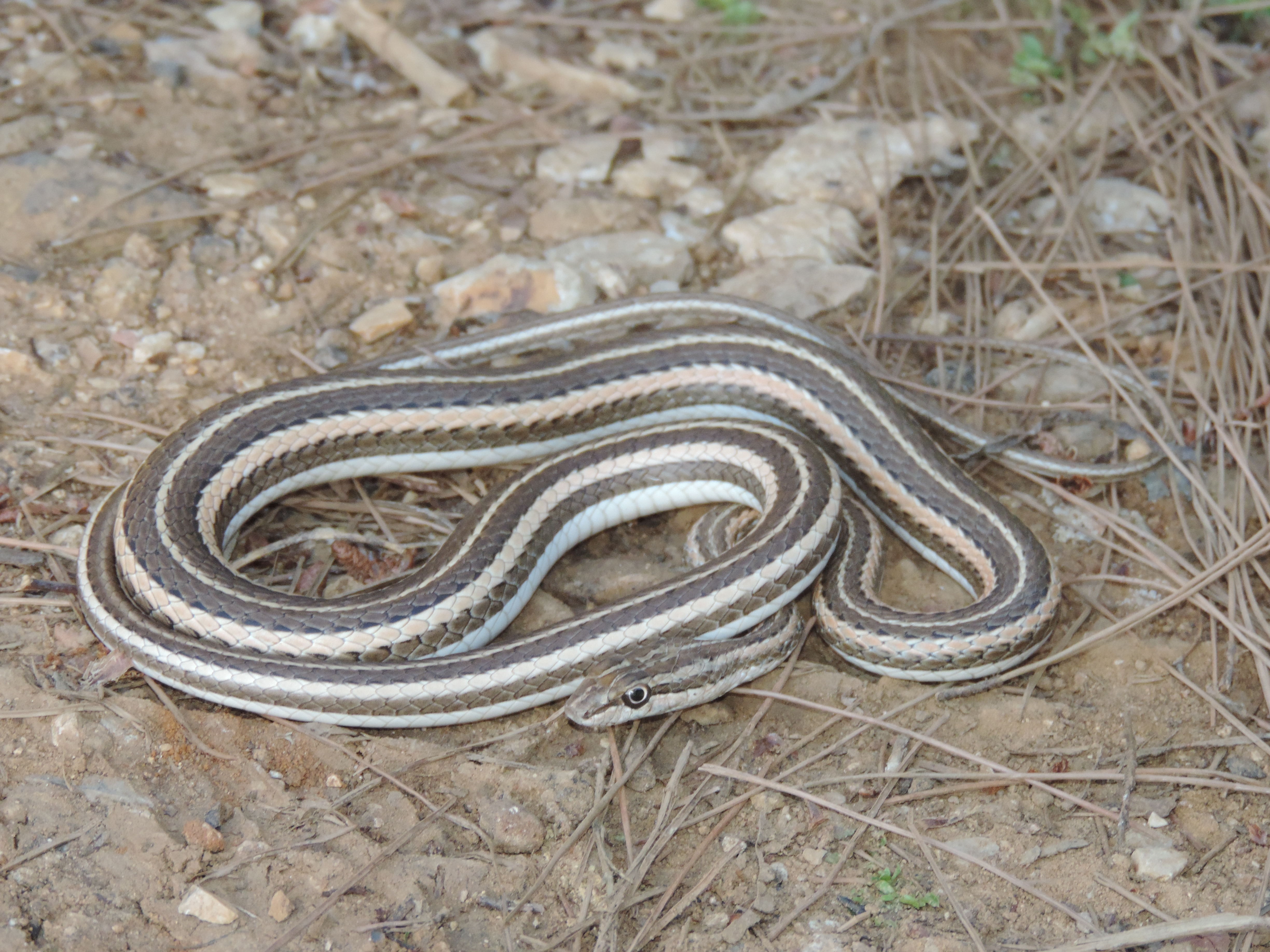

Supplement: Supplementary material 1 — Original photo vouchers of recorded individuals [file zookeys-1268-001_article-177920__-s001.zip › Supplementary_data/PSL011 (2).JPG]

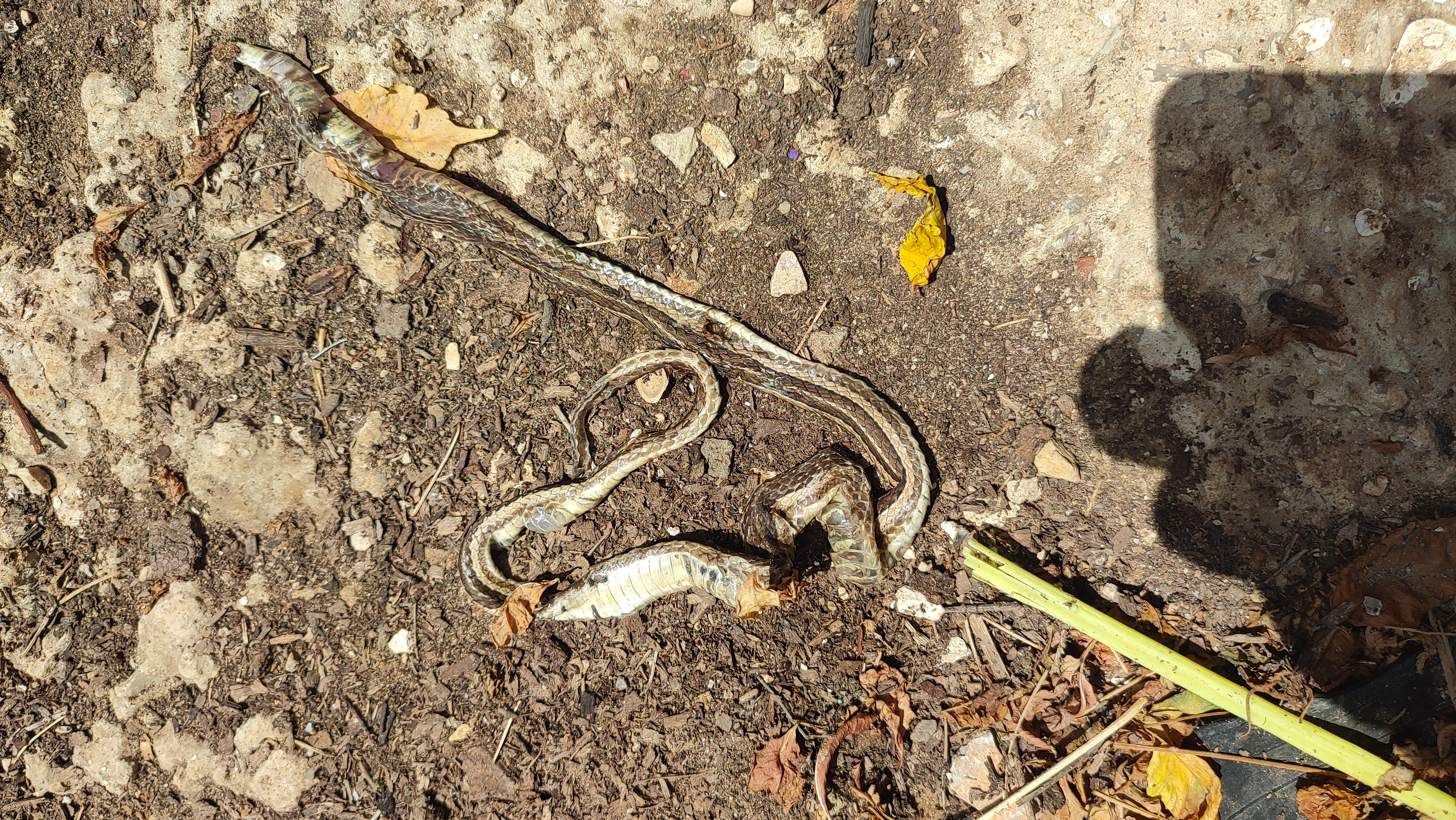

Supplement: Supplementary material 1 — Original photo vouchers of recorded individuals [file zookeys-1268-001_article-177920__-s001.zip › Supplementary_data/PSL012.jpg]

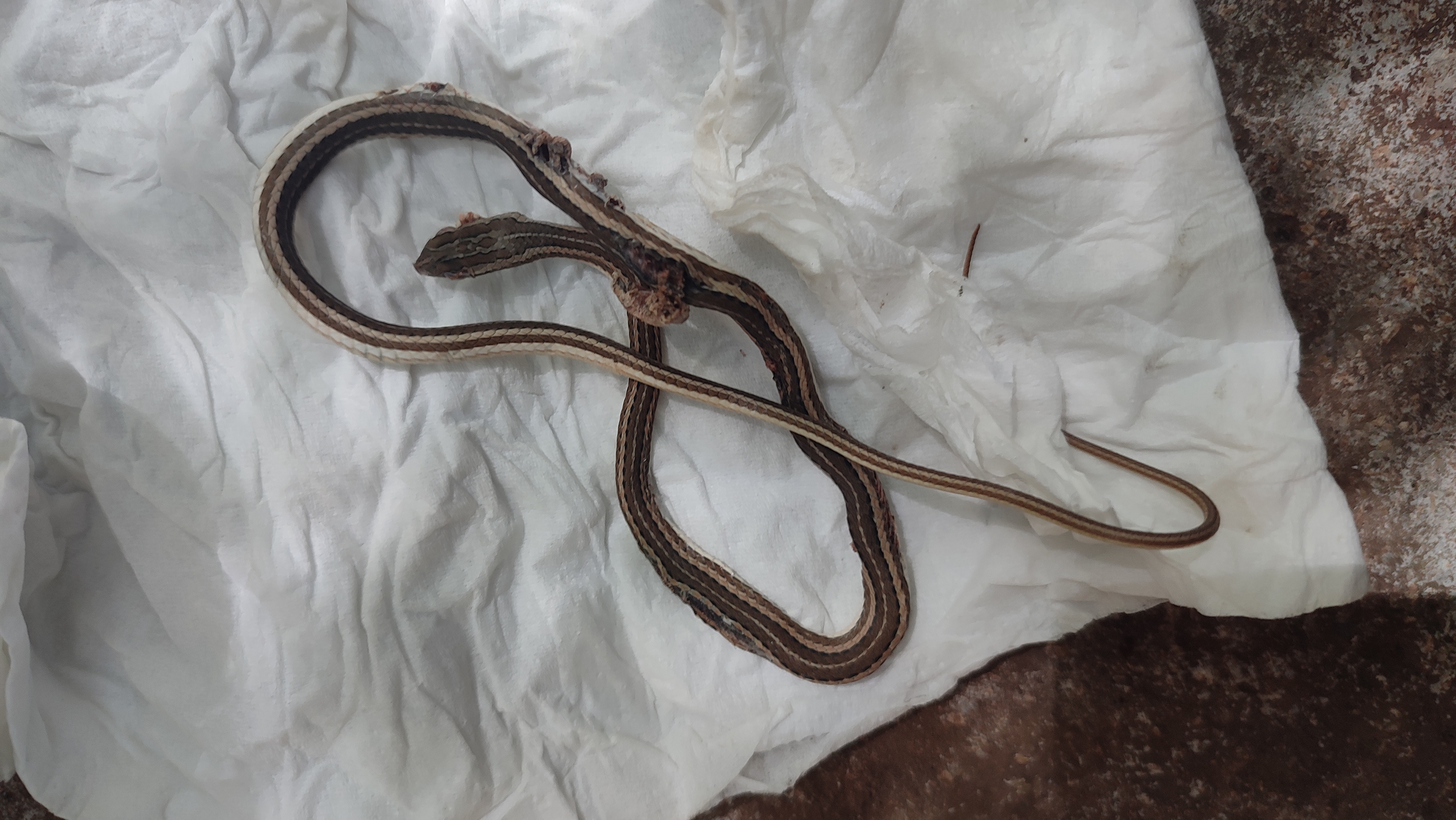

Supplement: Supplementary material 1 — Original photo vouchers of recorded individuals [file zookeys-1268-001_article-177920__-s001.zip › Supplementary_data/PSL013.jpg]

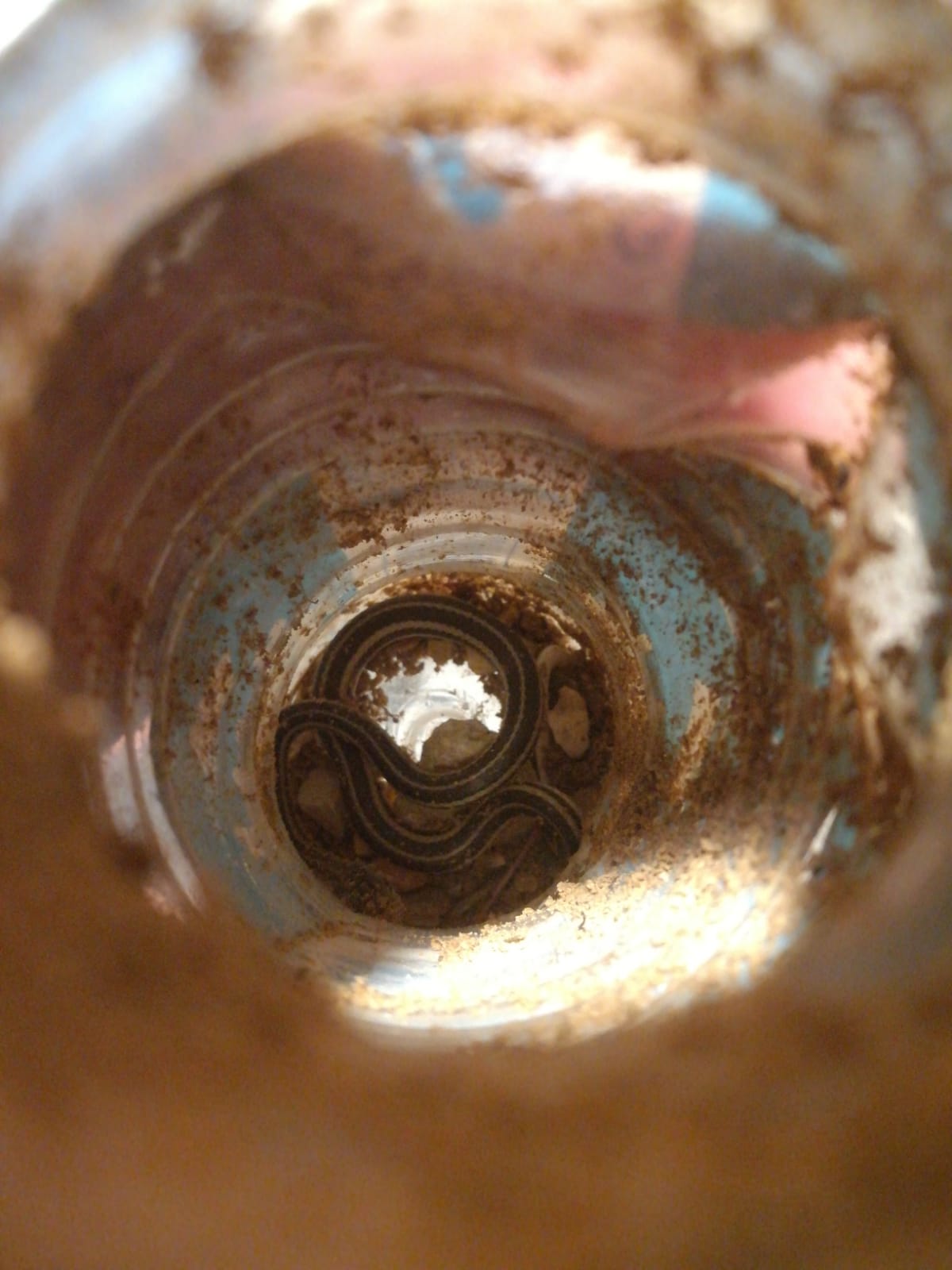

Supplement: Supplementary material 1 — Original photo vouchers of recorded individuals [file zookeys-1268-001_article-177920__-s001.zip › Supplementary_data/PSL014.jpg]

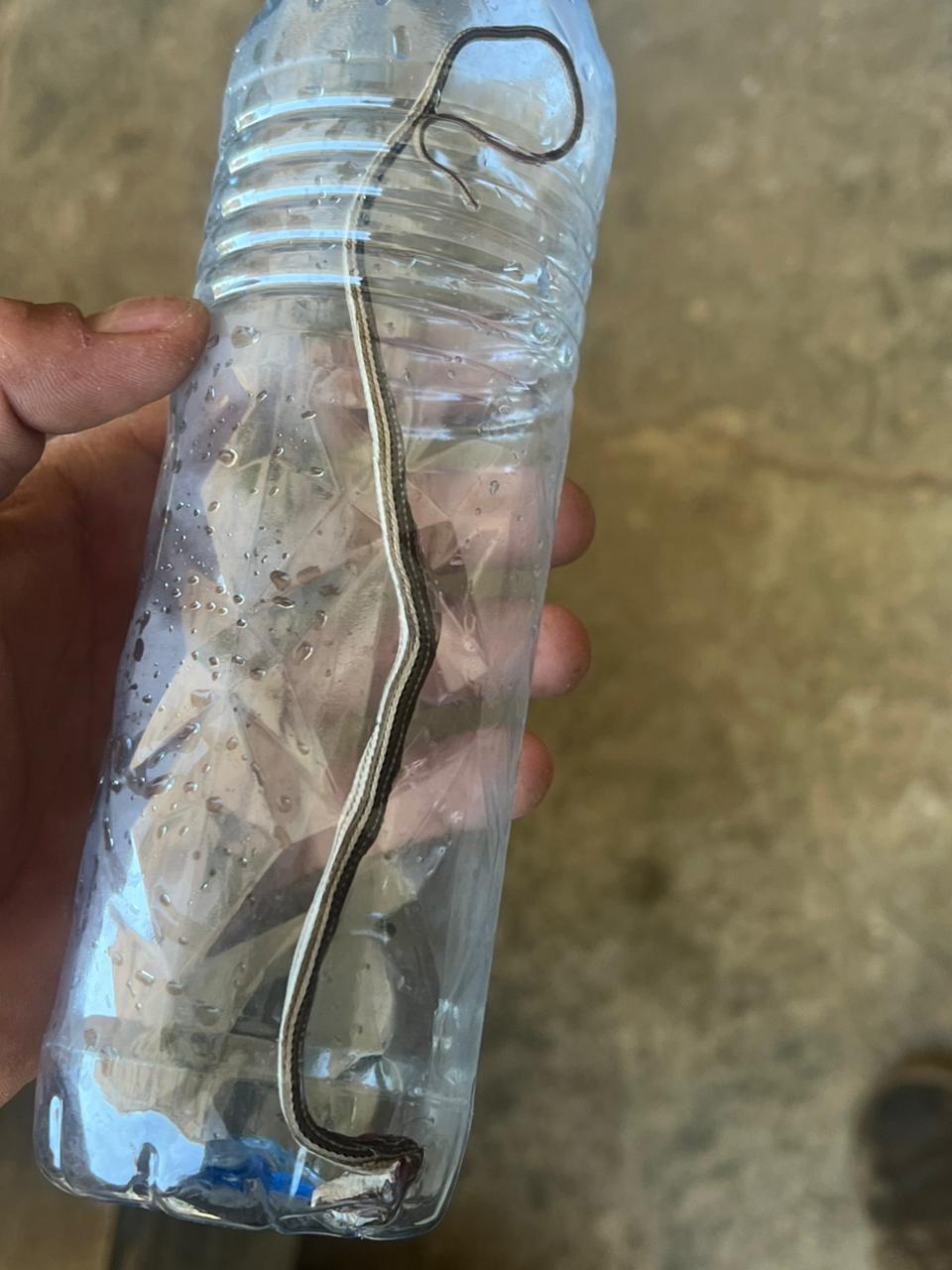

Supplement: Supplementary material 1 — Original photo vouchers of recorded individuals [file zookeys-1268-001_article-177920__-s001.zip › Supplementary_data/PSL015.jpg]

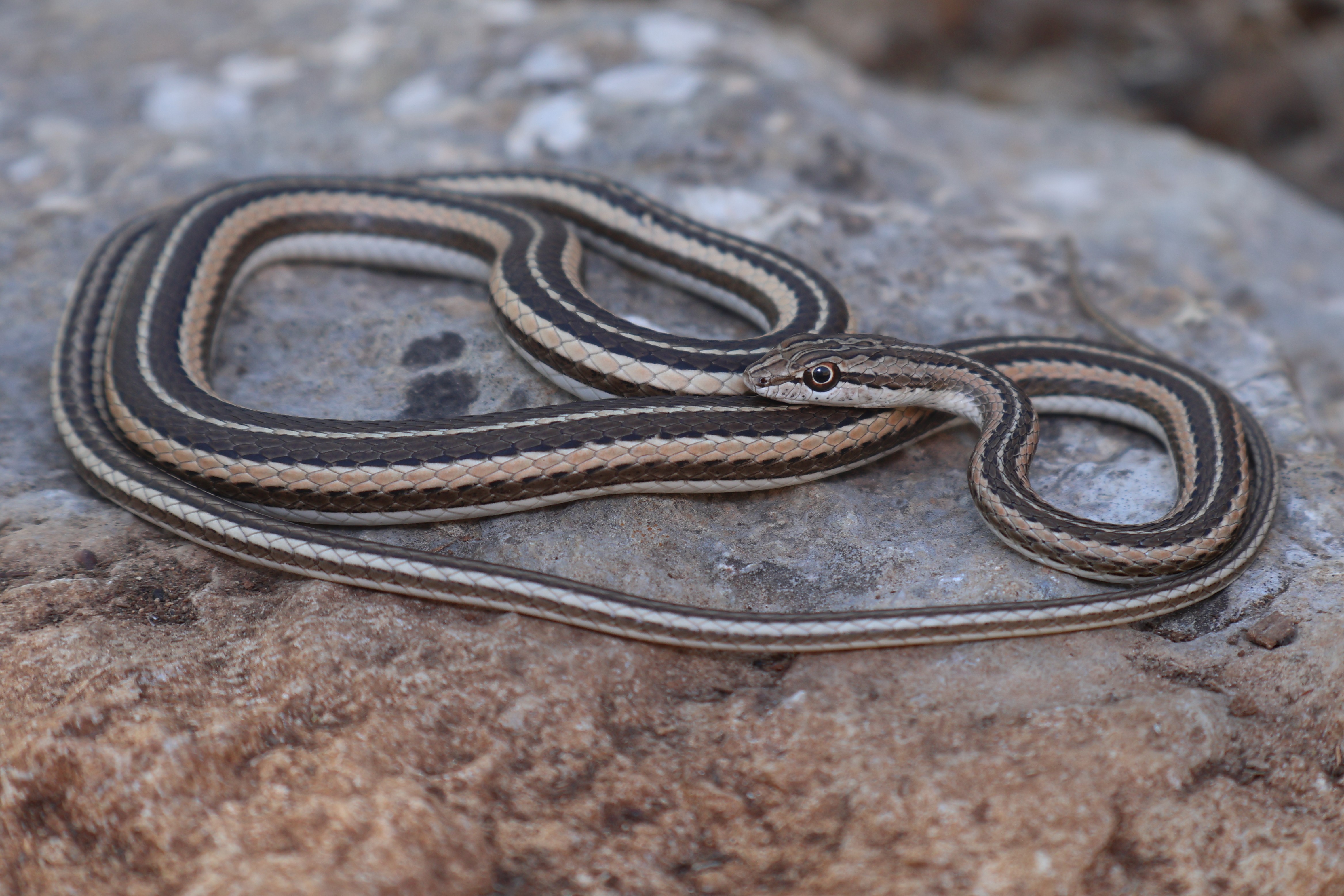

Supplement: Supplementary material 1 — Original photo vouchers of recorded individuals [file zookeys-1268-001_article-177920__-s001.zip › Supplementary_data/PSL016.JPG]
